# Interventions to prevent and treat sarcopenia in a surgical population: a systematic review and meta-analysis

**DOI:** 10.1093/bjsopen/zraa069

**Published:** 2021-06-24

**Authors:** S Tomassini, R Abbasciano, G J Murphy

**Affiliations:** Department of Cardiovascular Sciences and National Institute for Health Research Leicester Biomedical Research Unit in Cardiovascular Medicine, University of Leicester, Leicester, UK; Department of Cardiovascular Sciences and National Institute for Health Research Leicester Biomedical Research Unit in Cardiovascular Medicine, University of Leicester, Leicester, UK; Department of Cardiovascular Sciences and National Institute for Health Research Leicester Biomedical Research Unit in Cardiovascular Medicine, University of Leicester, Leicester, UK

## Abstract

**Background:**

The aim of this systematic review was to summarize the results of trials evaluating interventions for the reduction of sarcopenia in patients undergoing surgery.

**Methods:**

Searches were conducted using the Cochrane Central Register of Controlled Trials, MEDLINE and Embase. RCTs evaluating exercise, dietary or pharmacological interventions to address sarcopenia in the perioperative period were included. Treatment effect estimates were expressed as standardized mean differences (MDs) with confidence intervals, and heterogeneity was expressed as *I*^2^ values.

**Results:**

Seventy trials including 3402 participants were selected for the data synthesis. Exercise interventions significantly increased muscle mass (MD 0.62, 95 per cent c.i. 0.34 to 0.90; *P* < 0.001), muscle strength (MD 0.55, 0.39 to 0.71; *P* < 0.001), measures of gait speed (MD 0.42, 0.05 to 0.79; *P* = 0.03), and reduced time for completion of set exercises (MD −0.76, −1.12 to −0.40; *P* < 0.001) compared with controls. Subgroup analysis showed that interventions in the early postoperative period were more likely to have a positive effect on muscle mass (MD 0.71, 0.35 to 1.07; *P* < 0.001) and timed tests (MD −0.70, −1.10 to −0.30; *P* = 0.005) than preoperative interventions. Treatment effects on muscle mass (MD 0.09, −0.31 to 0.49; *P* = 0.66) and strength (MD 0.46, −0.01 to 0.92; *P* = 0.05) were attenuated by the presence of cancer. Results of analyses restricted to nine trials at low risk of allocation concealment bias and fourteen trials at low risk of attrition bias were comparable to those of the primary analysis. Risk**-**of**-**bias assessment showed that most trials were at high risk of incomplete outcome and attrition bias, thus reducing the estimate of certainty of the evidence according to the GRADE assessment tool.

**Conclusion:**

Exercise interventions appear beneficial in reducing the impact of sarcopenia. Because of the high risk of bias and low certainty of the current evidence, large RCTs using standardized measures of muscle mass should be undertaken.

## Teaser

This review has evaluated the available evidence from trials which have evaluated interventions for the reduction of sarcopenia in patients undergoing surgery. We have found that exercise interventions are likely to improve muscle strength and muscle mass as well as the quality of motor performance especially in the post-operative period. We have also found that the presence of cancer may attenuate the effect of certain interventions.

## Resumen

**Antecedentes:**

El objetivo de esta revisión sistemática fue resumir los resultados de los ensayos clínicos que evaluaron las intervenciones para reducir la sarcopenia en pacientes sometidos a cirugía.

**Métodos:**

Las búsquedas se realizaron mediante el Registro Central Cochrane de Ensayos Controlados, MEDLINE y EMBASE. Se incluyeron los ensayos aleatorizados y controlados (*randomised controlled trials*, RCT) que habían evaluado las intervenciones basadas en el ejercicio físico, dietéticas o farmacológicas para disminuir la sarcopenia en el período perioperatorio. Las estimaciones del efecto del tratamiento se expresaron como diferencias de medias estandarizadas (*standardised mean differences*, SMD) e intervalos de confianza, y la heterogeneidad se expresó como I^2^.

**Resultados:**

Se seleccionaron 70 ensayos con 3.402 participantes para la síntesis de los datos. Las intervenciones con ejercicio aumentaron significativamente la masa muscular (SMD 0,62; i.c. del 95%: 0,34-0,90**;** *P* < 0,0001), la fuerza muscular (SMD 0,55; i.c. del 95%: 0,39-0,71**;** *P* < 0,0001), las medidas de la velocidad de la marcha (SMD 0,42**;** i.c. del 95% 0,05-0,79**;** *P* = 0,02), y disminuyeron el tiempo para completar los ejercicios de la serie (SMD -0,75**;** i.c. del 95% -1,08 **–**0,43 *P* < 0,0001), en comparación con los controles. El análisis de subgrupos mostró que las intervenciones en el período postoperatorio temprano tenían más probabilidades de tener un efecto positivo sobre la masa muscular (SMD 0,71**;** i.c. del 95%: 0,35-1,07**;** *P* < 0,0001) y las pruebas cronometradas (SMD -0,70**;** i.c. del 95% - 1,10-0,30**;** *P* = 0,005 I^2^ = 59%) en comparación con las intervenciones preoperatorias. Los efectos del tratamiento sobre la masa muscular (SMD 0,09**;** i.c. del 95%: -0,31-0,49**;**  *P* = 0,66) y la fuerza (SMD 0,46**;** -0,01-0,92**;** *P* = 0,05), se atenuaron por la presencia de cáncer. Los resultados de los análisis restringidos a cuatro ensayos con bajo riesgo de sesgo de ocultación de la asignación o dos ensayos con bajo riesgo de sesgo de desgaste fueron comparables a los del análisis primario. La evaluación del riesgo de sesgo mostró que la mayoría de los ensayos tenían un alto riesgo de resultado incompleto y sesgo de desgaste, lo que reducía la estimación de la certeza de la evidencia según la herramienta de evaluación GRADE.

Conclusión: Las intervenciones con ejercicios parecen ser beneficiosas para reducir el impacto de la sarcopenia. Debido al alto riesgo de sesgo y la baja certeza de la evidencia actual, se deben realizar grandes ensayos aleatorizados y controlados que utilicen medidas estandarizadas de masa muscular.

## Introduction

Sarcopenia, characterized by progressive and generalized muscle loss, is observed in over 20–40 per cent of patients recovering from major surgery, where it has been associated with higher rates of complications in sarcopenic compared with non-sarcopenic patients (45 *versus* 15 per cent), higher in-hospital mortality rates (23 *versus* 4 per cent)^1^, and longer hospital stay^2^. Sarcopenia is associated with advancing age and frailty, but can occur in younger patients^3^ who have additional risk factors, including sedentary lifestyle, poor nutrition, chronic disease, and chronic inflammatory states^4^.

As the population ages, and the numbers of patients with frailty, multiple chronic conditions or cardiometabolic disease referred for surgery increases, sarcopenia will present an increasing challenge to clinicians and health systems. Research points towards a complex and multifactorial pathophysiology characterized by loss of mitochondrial function in skeletal muscle, chronic inflammatory changes, and exposure to oxidative stress^5^ that may be modified by exercise, diet or pharmacological interventions. The aim of this review was to summarize the results of randomized trials of interventions aiming to attenuate sarcopenia in people undergoing surgery. The secondary aim was to evaluate the effectiveness of these interventions across a range of important subgroups defined by intervention type, age, disease type including cancer, and timing of the intervention. Finally, the strengths and limitations of different definitions of sarcopenia when measuring treatment effects in clinical trials were evaluated.

## Methods

A systematic review of RCTs was performed according to the methods described in the *Cochrane Handbook for Systematic Reviews of Interventions*^6^. A protocol was registered prospectively on PROSPERO (CRD42020165325)^7^. The study adhered to PRISMA guidelines^8^.

### Study eligibility

Studies were included if they fulfilled the inclusion criteria of RCTs in which an intervention was used to prevent or reverse sarcopenic changes in adult patients (over 18 years old) in a surgical population. Trials were excluded if they reported retrospective or observational studies, or included a significant proportion of people (over 50 per cent) with neuromuscular or neurodegenerative disease, cachexia, or chronic inflammatory conditions. Abstracts were reviewed and were included only if they were of high quality and adhered to CONSORT reporting criteria; recent studies have shown discrepancies between the data presented in conference abstracts and subsequent full-text publications^9^, or even between the abstract and main article text^10^. Furthermore, as the adherence of abstracts to the CONSORT reporting criteria has been reported as suboptimal^11^, these were only included if they adhered to CONSORT reporting guidelines.

### Search methods

Electronic searches were conducted in the Cochrane Central Register of Controlled Trials, MEDLINE, and Embase using the following search terms: sarcopenia, muscle mass, dietary proteins, exercise therapy, testosterone or androgen or growth hormone and related terms. A full description of the search terms is available in *Appendix S1*. The final search was undertaken on 19 December 2019.

### Study selection

Title and abstract screening were carried out independently by two authors using the Rayyan QCRI web app (Qatar Computer Research Institute, Hamad Bin Khalifa University, Doha, Qatar). Selected references were managed using Endnote™ X9 (Clarivate Analytics, Philadelphia, Pennsylvania, USA). Full-text screening was carried out and the reference lists of included papers were also screened for suitable articles. Excluded studies and the reason for exclusion were recorded. Disagreements were resolved by discussion or, where this was not possible, by a third author.

### Assessment of risk of bias in included studies

Included trials were appraised using the Cochrane risk**-**of**-**bias tool version 8^12^. Two authors assessed each outcome of interest as being at either at low, high or unclear risk of bias for each domain. Disagreements were resolved as above.

### Data extraction

Data were extracted by two reviewers and managed using Excel™ 2016 (Microsoft, Redmond, Washington, USA). This included year, study type, setting, sample size, participant demographics, baseline characteristics, type of surgery, details of interventions, outcomes, and risk**-**of**-**bias assessments. The primary outcome of this review was measures of sarcopenia, evaluated either by functional tests or imaging. A large variety of measures were used in the included trials; direct measures of muscle mass, such as cross-sectional area of lumbar spine or quadriceps muscle assessed by direct imaging (dual-energy x-ray absorptiometry (DEXA), CT, MRI), and muscle strength, such as hand-grip strength or equivalent, were included. Furthermore, in view of the definition of sarcopenia as a decline in muscle quantity and quality, and in keeping with an international consensus guideline^3^, the measures of muscle function were included. These can be broadly split into two categories: functional assessments which record the time taken to complete a specific task such as the timed-get-up-and go (TUG) test and the sit-to-stand test, and time-based tests in which the numbers of metres walked or repetitions of an exercise were measured, such as the 6-minute-walk test (6MWT).

For the purposes of meta-analyses, these measures were grouped into four categories. Category A comprised measures of muscle mass, including appendicular skeletal muscle mass evaluated by DEXA, CT or MRI; whole-body skeletal muscle mass by DEXA, CT or MRI; mid-thigh cross-sectional area by CT or MRI; lumbar muscle cross-sectional area; bioelectrical impedance analysis; measurement of muscle thickness by ultrasonography; measurements of muscle volume by ultrasound imaging, CT or MRI; or measurement of skinfold thickness. Category B included measures of muscle strength, including hand-grip strength, quadriceps muscle strength, lower limb (any muscle group) resistance test, and upper limb (any muscle group) resistance test not including hand-grip strength. Category C comprised tests for the completion of set exercises including chair stand test (sit to stand), TUG test, and 10-m walk test. As these trials measure the time for completion of the exercises, beneficial treatment effects are negative in these trials. Category D consisted of repetition-based tests in which the number of metres walked or repetitions of an exercise in a set timeframe were assessed, including gait speed, 6MWT, and 30-s chair-rise test.

Secondary outcomes included self-reported quality of life, anaemia, mortality at 30 days, rates of readmission, duration of hospital stay, and rates of admission to a more intensive place of care either upon hospital discharge or from the previous place of residence.

Subgroup analysis was undertaken for type of surgery (orthopaedic, cardiothoracic, general, gynaecological, urological, bariatric, breast, transplant, and trauma), timing of the intervention (preoperative, perioperative, early postoperative, late postoperative), age of the participants (Aged 65 or under or aged over 65), cancer status of the participants.

### Statistical analysis

Standardized mean differences (MDs) with 95 per cent confidence intervals and *P* values were estimated for treatment effect measures as continuous outcomes using an inverse-variance random**-**effects method. Risk ratios with 95 per cent confidence intervals were estimated for dichotomous outcomes using the Mantel–Haenszel random**–**effects method. All analyses were carried out using Review Manager (RevMan) version 5.3 (The Nordic Cochrane Centre, Copenhagen, Denmark).

The heterogeneity of treatment effects was explored using a prespecified subgroup analysis for the following criteria: type of surgery, cancer status, age, and timing of interventions. The test for subgroup differences in the Cochrane software was used to identify significant treatment–subgroup interactions. Sensitivity analyses excluded studies with high risk of bias in two domains—allocation concealment and incomplete outcome data—as it was predicted that these would be the most likely sources of bias in this review. Heterogeneity within each meta-analysis was explored by using a χ^2^ test with significance set at a *P* value of 0.10, and was expressed as percentage heterogeneity due to variation rather than to chance (*I*^2^). An *I*^2^ value of 0–40 per cent indicated no or mild heterogeneity; 41–80 per cent indicated moderate heterogeneity; and over 80 per cent represented severe heterogeneity.

Publication bias for the primary outcome was assessed using funnel plots, where 10 or more studies contributed to an outcome. The quality of evidence was assessed using Grading of Recommendations Assessment, Development and Evaluation (GRADE) methodology in GRADEPRO GDT software (https://gdt.gradepro.org)^13^^,^^14^.

## Results

### Study characteristics

**Fig. 1:**
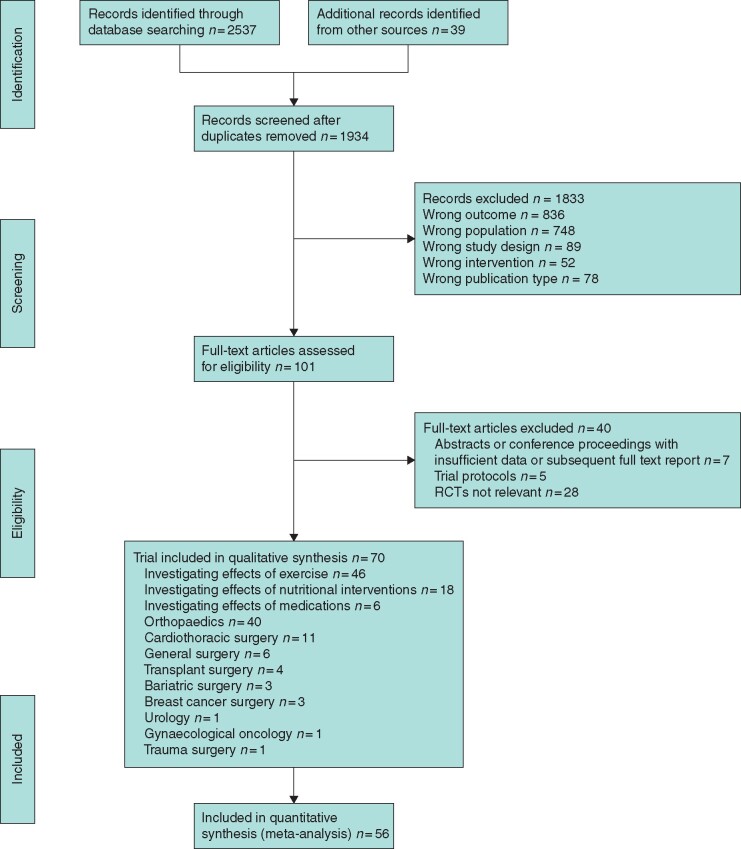
PRISMA diagram showing selection of studies for review

The results of the searches and exclusions are shown in *Fig. 1*. In total, 70 trials with 3402 participants (mean age 54.6 years), were included in the data synthesis (*Table S1*)^15–73^. These included six trials^18^^,^^38^^,^^41^ in general surgery, three^34^^,^^54^^,^^59^ in bariatric surgery, three^15^^,^^17^ in breast cancer surgery, eleven^16^^,^^23^^,^^31^^,^^32^^,^^47^^,^^48^^,^^50^^,^^65^^,^^70^^,^^73^ in cardiothoracic surgery, forty-one^19–21^^,^^24^^,^^26–30^^,^^33^^,^^35–37^^,^^39^^,^^40^^,^^43^^,^^45^^,^^49^^,^^51–53^^,^^55^^,^^57^^,^^58^^,^^60–64^^,^^66–69^^,^^71^^,^^72^ in orthopaedics, three^42^^,^^44^^,^^46^ in transplant surgery, one^56^ in urology, one^25^ in gynaecological surgery, and one^22^ in trauma surgery. Some papers reported results of trial with multiple treatment arms: for the purpose of the meta-analysis, each treatment arm has been considered as a separate trial. The average age of the participants 65 or less in thirty-nine trials and over 65 years in twenty-six trials. Five trials did not report the average age of participants. There were eleven trials involving oncological patients. With regard to timings of intervention, four trials investigated preoperative interventions (interventions introduced at any time before operation), five trials perioperative interventions (interventions introduced before operation and continued in postoperative period), forty-seven trials investigated early postoperative intervention (interventions started within first 6 weeks after surgery) and fourteen trials late postoperative interventions (interventions started 6 weeks after operation). Forty-six trials investigated the effects of exercise, eighteen studied nutritional interventions, and six the effects of medications. A summary of the main findings of interventions are reported in *Table 1*.

**Table 1:** Summary of findings with trial interventions for primary and secondary outcomes including intragroup and intergroup heterogeneity

	No. of studies	No. of participants	Treatment effect		Heterogeneity	
			Effect size	*P*	*I* ^2^ (%)	*P*
**Category A: measures of muscle mass**						
Overall effect	24	852	MD 0.48 (0.25, 0.70)	< 0.001	54	< 0.001
Exercise	17	606	MD 0.62 (0.34, 0.90)	< 0.001	55	0.004
Dietary interventions	5	210	MD –0.01 (–0.28, 0.27)	0.96	0	0.42
Medications	2	36	MD 0.87 (0.18, 1.57)	0.01	0	0.38
Heterogeneity between subgroups					83.8	0.002
**Category B: measures of muscle strength**						
Overall effect	21	793	MD 0.49 (0.35, 0.63)	< 0.001	0	0.46
Exercise	18	670	MD 0.55 (0.39, 0.71)	< 0.001	0	0.51
Dietary intervention	3	123	MD 0.19 (–0.17, 0.55)	0.30	0	0.75
Medications	0	0	Not estimable	Not estimable	Not estimable	
Heterogeneity between subgroups					69.5	0.07
**Category C: timed tests**						
Overall effect	9	432	MD –0.75 (–1.08, –0.43)	< 0.001	50	0.04
Exercise	8	411	MD –0.76 (–1.12, –0.40)	< 0.001	56	0.02
Dietary interventions	1	21	MD –0.76 (–1.66, 0.14)	0.10	Not estimable	
Medications	0	0	Not estimable	Not estimable	Not estimable	
Heterogeneity between subgroups					0	1.00
**Category D: repetition-based tests**						
Overall effect	10	631	MD 0.35 (0.06, 0.64)	0.02	51	0.03
Exercise	9	490	MD 0.42 (0.05, 0.79)	0.03	55	0.02
Dietary interventions	1	141	MD 0.13 (–0.20, 0.46)	0.44	Not estimable	
Medications	0	0	Not estimable	Not estimable	Not estimable	
Heterogeneity between subgroups					24.3	0.25
**Self-reported quality of life**						
Overall effect	13	855	MD 0.28 (0.10, 0.45)	0.002	30	0.15
Exercise	11	719	MD 0.27 (0.08, 0.47)	0.006	32	0.14
Dietary interventions	1	101	MD 0.49 (0.09, 0.88)	0.02	Not estimable	
Medications	1	35	MD –0.10 (–0.80, 0.60)	0.78	Not estimable	
Heterogeneity between subgroups					7.5	0.34
**Discharge to higher level of care**						
Overall effect	2	139	OR 0.36 (0.13, 1.04)	0.06	0	0.96
Exercise	2	139	OR 0.36 (0.13, 1.04)	0.06	0	0.96
Dietary interventions	0	0	Not estimable	Not estimable	Not estimable	
Medications	0	0	Not estimable	Not estimable	Not estimable	
Heterogeneity between subgroups					Not estimable	
**Mortality at 30 days**						
Overall effect	2	102	OR 0.38 (0.07, 2.21)	0.28	0	0.41
Exercise	0	0	Not estimable	Not estimable	Not estimable	
Dietary interventions	1	61	OR 0.97 (0.06, 16.19)	0.98	Not estimable	
Medications	1	41	OR 0.21 (0.02, 2.00)	0.18	Not estimable	
Heterogeneity between subgroups					0	0.41
**Readmission rates**						
Overall effect	3	113	OR 0.36 (0.10, 1.31)	0.12	0	0.95
Exercise	1	32	OR 0.47 (0.04, 5.73)	0.55	Not estimable	
Dietary interventions	1	61	OR 0.34 (0.06, 1.94)	0.23	Not estimable	
Medications	1	20	OR 0.25 (0.01, 6.82)	0.41	Not estimable	
Heterogeneity between subgroups					0	0.95
**Duration of hospital stay**						
Overall effect	17	1154	MD –0.34 (–0.70, 0.01)	0.06	87	<0.00001
Exercise	9	632	MD –0.70 (–1.30, –0.10)	0.02	90	<0.00001
Dietary interventions	7	481	MD –0.03 (–0.20, 0.15)	0.78	0	0.97
Medications	1	41	MD 0.06 (–0.56, 0.67)	0.85	Not estimable	
Heterogeneity between subgroups					57.2	0.10

Values in parentheses are 95 per cent confidence intervals. MD, mean difference; OR, odds ratio.

### Assessment of bias

The results of the risk**-**of**-**bias assessments are shown in *Fig. S1*. Overall, the methodological quality of the included trials was low. A total of nine trials were at low risk of allocation concealment and fourteen trials were considered to be at low risk of attrition bias.

### Data synthesis

For the primary analyses, the studies were grouped by one of the four methods of sarcopenia assessment. In addition, the treatment effect was also determined for each of the three therapeutic approaches to sarcopenia: exercise interventions, dietary interventions, and pharmacological interventions. Twenty-four trials were included in category A (quantitative measures of muscle mass), 21 in category B (measures of muscle strength), nine in category C (timed tests), and 10 in category D (repetition-based tests).

**Fig. 2:**
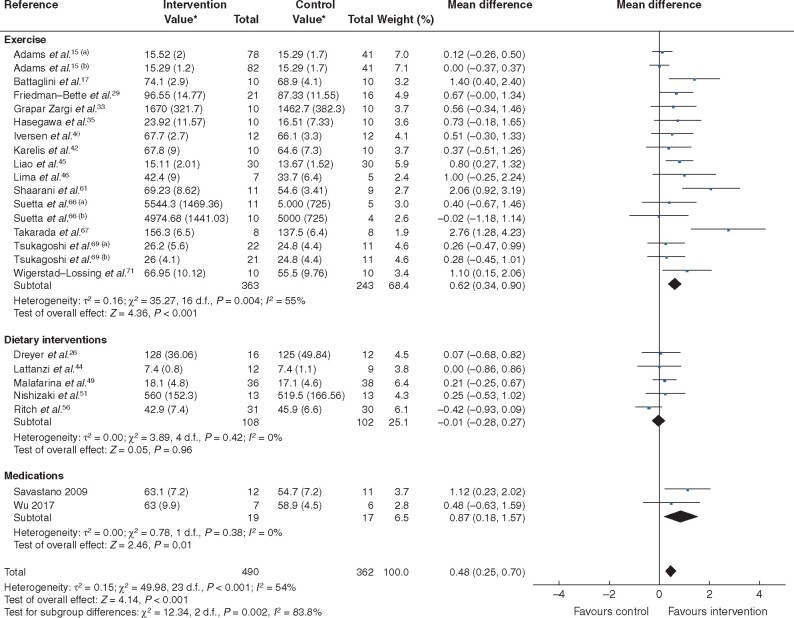
Forest plot of effects of interventions on category A measures (measures of muscle mass) with further subgroup analysis by category of intervention (exercise, dietary intervention or medications) An inverse-variance random-effects model was used for meta-analysis. Mean differences are shown with 95 per cent confidence intervals. *Values are mean(s.d.). Separate trials within a single publication are referred to as a and b.

Trial interventions (24 trials, 852 patients) improved quantitative measures of muscle mass (MD 0.48, 95 per cent c.i. 0.25 to 0.70; *P* < 0.001; *I*^2^ = 54 per cent) (*Fig. 2* and *Table 1*). When stratified by type of treatment, exercise (17 trials, 606 patients; MD 0.62, 0.34 to 0.90; *P* < 0.001; *I*^2^ = 55 per cent) and pharmacological strategies (2 trials, 36 patients; MD 0.87, 0.18 to 1.57; *P* = 0.01; *I*^2^ = 0 per cent) increased muscle mass, whereas dietary interventions did not (5 trials, 210 patients; MD −0.01, −0.28 to 0.27; *P* = 0.96; *I*^2^ = 0 per cent).

**Fig. 3:**
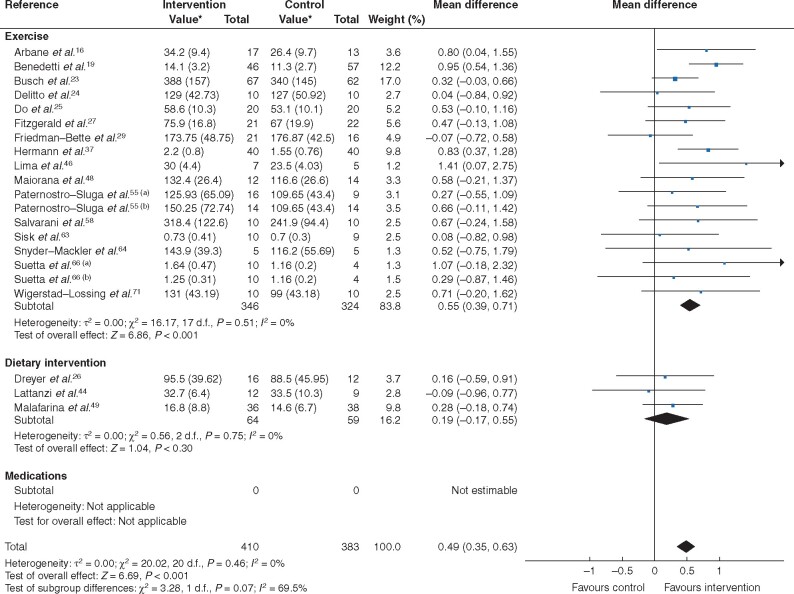
Forest plot of effects of interventions on category B measures (measures of muscle strength) with further subgroup analysis by category of intervention (exercise, dietary intervention or medications) An inverse-variance random-effects model was used for meta-analysis. Mean differences are shown with 95 per cent confidence intervals. *Values are mean(s.d.). Separate trials within a single publication are referred to as a and b.

Trial interventions (21 trials, 793 patients) improved measures of muscle strength (MD 0.49, 0.35 to 0.63; *P* < 0.001; *I*^2^ = 0 per cent), without heterogeneity (*Fig. 3* and *Table 1*). In analyses stratified by type of treatment, exercise significantly increased muscle strength (18 trials, 670 patients; MD 0.55, 0.39 to 0.71; *P* < 0.001; *I*^2^ = 0 per cent), whereas dietary interventions did not (3 trials, 123 patients; MD 0.19, −0.17 to 0.55; *P* = 0.30; *I*^2^ = 0 per cent). No pharmacological trials reported these outcomes.

**Fig. 4:**
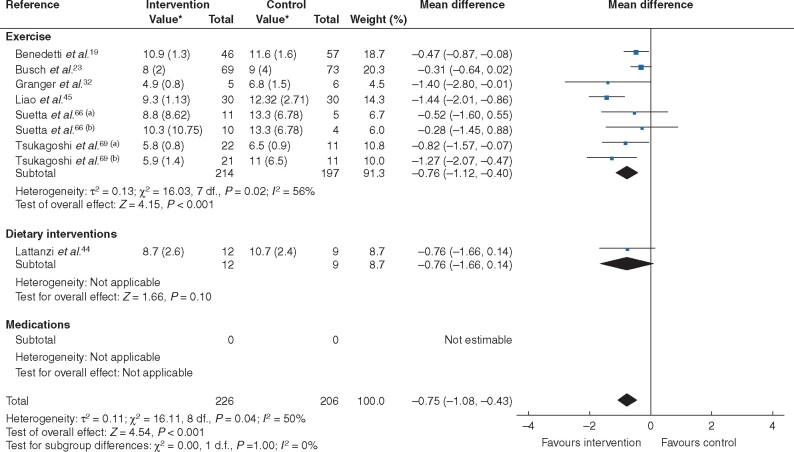
Forest plot of effects of interventions on category C measures (time needed for completion of a set number of exercises) with further subgroup analysis by category of intervention (exercise, dietary intervention or medications) An inverse-variance random-effects model was used for meta-analysis. Mean differences are shown with 95 per cent confidence intervals. *Values are mean(s.d.). Separate trials within a single publication are referred to as a and b.

Trial interventions (9 trials, 432 patients) reduced times for completion of sit–stand and other similar tests (MD −0.75, −1.08 to −0.43; *P* < 0.001; *I*^2^ = 50 per cent) with moderate heterogeneity (*Fig. 4* and *Table 1*). In analyses stratified by type of treatment, exercise significantly reduced test times (9 trials, 411 patients; MD −0.76, −1.12 to −0.40; *P* < 0.001; *I*^2^ = 56 per cent), whereas dietary interventions did not (1 trial, 21 patients; MD −0.76, −1.16 to 0.14; *P* = 0.30). No pharmacological trials reported these outcomes.

**Fig. 5:**
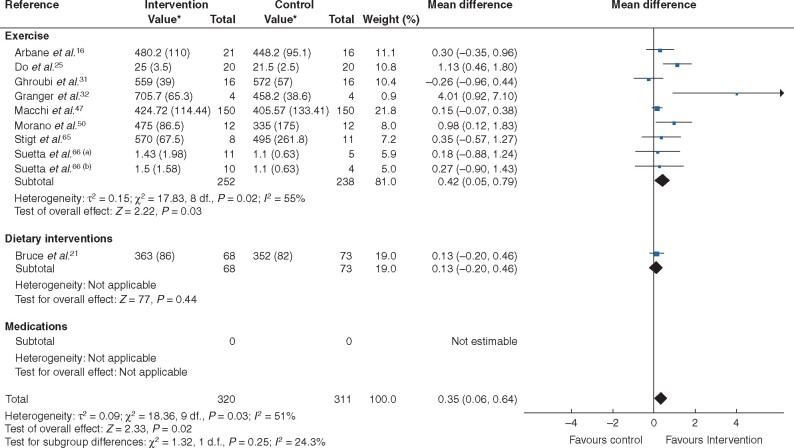
Forest plot of effects of interventions on category D measures (number of repetitions completed in a set time interval in repetition-based tests) with further subgroup analysis by category of intervention (exercise, dietary intervention or medications) An inverse-variance random-effects model was used for meta-analysis. Mean differences are shown with 95 per cent confidence intervals. *Values are mean(s.d.). Separate trials within a single publication are referred to as a and b.

Trial interventions (10 trials, 631 patients) improved measures of walking speed (MD 0.35, 0.06 to 0.64; *P* = 0.02; *I*^2^ = 51 per cent) with moderate heterogeneity (*Fig. 5* and *Table 1*). When stratified by type of treatment, exercise increased walking speed (9 trials, 490 patients; MD 0.42, 0.05 to 0.79; *P* = 0.03; *I*^2^ = 55 per cent), whereas dietary interventions did not (1 trial, 141 patients; MD 0.13, −0.20 to 0.46; *P* = 0.44). No pharmacological trials reported these outcomes.

Other treatment effects of interventions overall, and stratified by each of three main intervention groups are reported in *Table 1*. In meta-analyses, trial interventions (13 trials, 855 patients) led to an improvement in self-reported quality of life (MD 0.28, 0.10 to 0.45; *P* = 0.002; *I*^2^ = 30 per cent), with a similar treatment effect estimate for exercise trials (11 trials, 719 patients) and one trial (101 patients) of dietary intervention, but not in one other trial (35 patients) of a pharmacological intervention. Trial interventions (17 trials, 1154 patients) resulted in a non-statistically significant reduction in hospital stay (MD −0.34, −0.70 to 0.01; *P* = 0.06; *I*^2^ = 87 per cent) with severe heterogeneity. For duration of stay there was a significant treatment effect for exercise trials, but not for trials of dietary interventions, or for one trial of a pharmacological intervention (*Table 1*). Summary treatment estimates for other secondary outcomes were limited by small numbers of trials reporting each outcome (*Table 1*). No data were reported on levels of anaemia after surgery.

### Subgroup analyses

The results of subgroup analyses for the primary outcome are reported in *Table S2*. The subgroups analysed by type of surgery were limited by small numbers of trials in all specialties except orthopaedic surgery, where treatment effects were similar to those for the primary analysis: category A (16 trials, 453 patients; MD 0.57, 95 per cent c.i. 0.32 to 0.83; *P* < 0.001; *I*^2^ = 38 per cent); category B (12 trials, 472 patients; MD 0.49, 0.27 to 0.72; *P* < 0.001; *I*^2^ =22 per cent); category C (6 trials, 258 patients; MD −0.84, −1.25 to −0.44; *P* < 0.001; *I*^2^ = 49 per cent), and category D (3 trials, *n* = 171 patients; MD 0.14, −0.16 to 0.45; *P* = 0.35; *I*^2^ = 0 per cent). There was no treatment–subgroup interaction for any of the four measures of the primary outcome for age.

For timing of the intervention (preoperative, perioperative, early postoperative, late postoperative) there was a treatment–subgroup interaction with significant treatment effects for interventions in the early postoperative period aimed at increasing muscle mass (4 trials, 308 patients; MD 0.71, 0.35 to 1.07; *P* < 0.001; *I*^2^ = 49 per cent) and timed tests (7 trials, 367 patients; MD −0.70, −1.10 to −0.30; *P* = 0.005; *I*^2^ = 59 per cent). There was also a treatment–subgroup interaction with significant treatment effects for late postoperative interventions aimed at reducing timed-test scores (2 trials, 65 patients; MD −0.64, −1.17 to −0.11; *P* = 0.02; *I*^2^ = 0 per cent).

There was a significant interaction with cancer status and treatment effects, with no significant treatment effect on muscle mass among patients with cancer (5 trials, 344 patients; MD 0.09, −0.31 to 0.49; *P* = 0.66; *I*^2^ = 62 per cent), whereas there was a significant benefit in patients without cancer (17 trials, 472 patients; MD 0.58, 0.33 to 0.83; *P* < 0.001; *I*^2^ = 36 per cent). Similarly, patients with cancer showed no improvement in muscle strength (3 trials, 91 patients; MD 0.46, −0.01 to 0.92; *P* = 0.05; *I*^2^ = 16 per cent), whereas those without cancer did (18 trials, 702 patients; MD 0.49, 0.34 to 0.65; *P* < 0.001; *I*^2^ = 4 per cent). Such interactions were not seen for timed tests (no significant interaction) or walking speed; for the latter, there was a significant benefit in patients with cancer (5 trials, 128 patients; MD 0.80, 0.22 to 1.39; *P* = 0.007; *I*^2^ = 52 per cent), but not for patients without cancer (5 trials, 503 patients; MD 0.12, −0.05 to 0.30; *P* = 0.17; *I*^2^ = 0 per cent). Subgroup analyses for secondary outcomes are reported in *Table S3*.

### Sensitivity analysis

Sensitivity analyses for the primary outcome (*Table S4*) restricted to trials with adequate allocation concealment demonstrated a significant treatment effect on muscle mass (4 trials, 322 patients; MD 0.43, 95 per cent c.i. 0.03 to 0.83; *P* = 0.03; *I*^2^ = 91 per cent), muscle strength (2 trials, 209 patients; MD 0.55, 0.05 to 1.55; *P* = 0.03; *I*^2^ = 67 per cent), and for walking speed (1 trial, 24 patients; MD 0.98, 0.12 to 1.83; *P* = 0.02), but not for timed tests (2 trials, 202 patients; MD −0.85, −1.95 to 0.25; *P* = 0.13).

Sensitivity analyses restricted to trials at low risk of attrition bias demonstrated beneficial treatment effects for muscle mass (9 trials, 305 patients; MD 0.60, 0.14 to 1.06; *P* = 0.01; *I*^2^ = 71 per cent), and timed tests (4 trials, 228 patients; MD −0.96, −1.48 to −0.45; *P* = 0.002; *I*^2^ = 65 per cent), but not for muscle strength (4 trials, 170 patients; MD 0.40, −0.21 to 1.00; *P* = 0.20; *I*^2^ = 64 per cent), or walking speed (2 trials, 441 patients; MD 0.15, −0.04 to 0.33; *P* = 0.13; *I*^2^ = 0 per cent) (*Table S4*).

### GRADE assessments

GRADE assessment judged the certainty of the effect estimates for exercise interventions on muscle mass or strength as low, and those for timed walking tests, or gait speed or equivalent assessments as moderate (*Tables 2–5*). The certainty of the effect estimates for pharmacological or dietary interventions were low or very low for all measures of sarcopenia.

**Table 2:** Summary of findings for primary and secondary outcomes for interventions aimed at treating or preventing sarcopenia, with summary of GRADE assessment of reliability of available evidence

Certainty assessment						No. of patients*		Effect^†^		Certainty	Importance
No. of studies	Risk of bias	Inconsistency	Indirectness	Imprecision	Other considerations	Interventions	Standard management	Relative	Absolute		
**Quantitative measures of muscle mass**											
24 RCTs	Serious	Not serious	Not serious	Not serious	Publication bias strongly suspected	490	362		MD 0.48 (0.25, 0.70) higher	⊕⊕◯◯ Low	Important
**Quantitative measures of muscle strength**											
21 RCTs	Serious	Not serious	Not serious	Not serious	Publication bias strongly suspected	410	383		MD 0.49 (0.35, 0.63) higher	⊕⊕◯◯ Low	Important
**Timed tests**											
9 RCTs	Serious	Not serious	Not serious	Not serious	None	226	206		MD 0.75 (1.08, 0.43) lower	⊕⊕⊕◯ Moderate	Important
**Repetition-based tests**											
10 RCTs	Serious	Not serious	Not serious	Not serious	None	320	311		MD 0.35 (0.06, 0.64) higher	⊕⊕⊕◯ Moderate	Important
**Self-reported quality of life**											
13 RCTs	Very serious	Not serious	Not serious	Not serious	None	479	376		MD 0.28 (0.10, 0.45) higher	⊕⊕◯◯ Low	Important
**Mortality at 30 days**											
2 RCTs	Not serious	Not serious	Not serious	Serious	None	2 of 49 (4)	6 of 53 (11)	OR 0.38 (0.07 to 2.21)	67 fewer (from 104 fewer to 107 more) per 1000	⊕⊕⊕◯ Moderate	Important
**Duration of hospital stay**											
17 RCTs	Serious	Not serious	Not serious	Serious	None	579	575		MD 0.34 lower (from 0.70 lower to 0.01 higher)	⊕⊕◯◯ Low	Important

Values in parentheses are *percentages and ^†^95 per cent confidence intervals. GRADE, Grading of Recommendations Assessment, Development and Evaluation; MD, mean difference; OR, odds ratio.

**Table 3:** Summary of GRADE assessment of available evidence investigating effects of exercise interventions on all measures of sarcopenia and on prespecified secondary outcomes

Certainty assessment						No. of patients*		Effect^†^		Certainty	Importance
No. of studies	Risk of bias	Inconsistency	Indirectness	Imprecision	Other considerations	Exercise interventions	Standard management	Relative	Absolute		
**Quantitative measures of muscle mass**											
17 RCTs	Serious	Not serious	Not serious	Not serious	Publication bias strongly suspected	363	243		MD 0.62 (0.34, 0.90) higher	⊕⊕◯◯ Low	Important
**Quantitative measures of muscle strength**											
18 RCTs	Serious	Not serious	Not serious	Not serious	Publication bias strongly suspected	346	324		MD 0.55 (0.39, 0.71) higher	⊕⊕◯◯ Low	Important
**Timed tests**											
8 RCTs	Serious	Not serious	Not serious	Not serious	None	214	197		MD 0.76 (1.12, 0.40) lower	⊕⊕⊕◯ Moderate	Important
**Repetition-based tests**											
9 RCTs	Serious	Not serious	Not serious	Not serious	None	252	238		MD 0.42 (0.05, 0.79) higher	⊕⊕⊕◯ Moderate	Important
**Self-reported quality of life**											
11 RCTs	Very serious	Not serious	Not serious	Not serious	None	404	315		MD 0.27 (0.08, 0.47) higher	⊕⊕◯◯ Low	Important
**Discharge to higher level of care**											
2 RCTs	Very serious	Not serious	Not serious	Serious	None	5 of 69 (7)	13 of 70 (19)	OR 0.36 (0.13, 1.04)	110 fewer (from 157 fewer to 6 more) per 1000	⊕◯◯◯ Very low	Critical
**Duration of hospital stay - exercise**											
9 RCTs	Serious	Not serious	Not serious	Not serious	Publication bias strongly suspected	324	308		MD 0.7 (1.30, 0.10) lower	⊕⊕◯◯ Low	Critical

Values in parentheses are *percentages and ^†^95 per cent confidence intervals. GRADE, Grading of Recommendations Assessment, Development and Evaluation; MD, mean difference; OR, odds ratio.

**Table 4:** Summary of GRADE assessment of available evidence investigating effects of dietary interventions on all measures of sarcopenia and on prespecified secondary outcomes

Certainty assessment						No. of patients*		Effect^†^		Certainty	Importance
No. of studies	Risk of bias	Inconsistency	Indirectness	Imprecision	Other considerations	Dietary interventions	Standard management	Relative	Absolute		
**Quantitative measures of muscle mass**											
5 RCTs	Serious	Not serious	Not serious	Serious	None	108	102		MD 0.01 lower (from 0.28 lower to 0.27 higher)	⊕⊕◯◯ Low	Important
**Quantitative measures of muscle strength**											
3 RCTs	Serious	Not serious	Not serious	Serious	None	64	59		MD 0.19 higher (from 0.17 lower to 0.55 higher)	⊕⊕◯◯ Low	Important
**Timed tests**											
1 RCT	Very serious	Not serious	Not serious	Very serious	None	12	9		MD 0.76 lower (from 1.66 lower to 0.14 higher)	⊕◯◯◯ Very low	Important
**Repetition-based tests**											
1 RCT	Very serious	Not serious	Not serious	Very serious	None	68	73		MD 0.13 higher (from 0.20 lower to 0.46 higher)	⊕◯◯◯ Very low	Important
**Self-reported quality of life: dietary interventions**											
1 RCT	Very serious	Not serious	Not serious	Serious	None	52	49		MD 0.49 (0.09, 0.88) higher	⊕◯◯◯ Very low	Important
**Mortality at 30 day: dietary interventions**											
1 RCT	Not serious	Not serious	Not serious	Very serious	None	1 of 31 (3)	1 of 30 (3)	OR 0.97 (0.06, 16.19)	1 fewer (from 31 fewer to 325 more) per 1000	⊕⊕◯◯ Low	Critical
**Readmission rates: dietary interventions**											
1 RCT	Not serious	Not serious	Not serious	Very serious	None	2 of 31 (6)	5 of 30 (17)	OR 0.34 (0.06, 1.94)	103 fewer (from 155 fewer to 113 more) per 1000	⊕⊕◯◯ Low	Important
**Duration of hospital stay: dietary interventions**											
7 RCTs	Serious	Not serious	Not serious	Serious	None	237	244		MD 0.03 lower (from 0.20 lower to 0.15 higher)	⊕⊕◯◯ Low	Critical

Values in parentheses are *percentages and ^†^95 per cent confidence intervals. GRADE, Grading of Recommendations Assessment, Development and Evaluation; MD, mean difference; OR, odds ratio.

**Table 5:** Summary of the GRADE assessment of available evidence investigating effects of pharmacological interventions on all measures of sarcopenia and on prespecified secondary outcomes

Certainty assessment							No. of patients*		**Effect** ^†^		Certainty	Importance
No. of studies	Study design	Risk of bias	Inconsistency	Indirectness	Imprecision	Other considerations	Medications	Standard management	Relative	Absolute		
**Quantitative measures of muscle mass**												
2 RCTs		Serious	Not serious	Not serious	Serious	None	19	17		MD 0.87 (0.18, 1.57) higher	⊕⊕◯◯ Low	Important
**Self-reported quality of life: medications**												
1 RCT		Serious	Not serious	Not serious	Serious	None	23	12		MD 0.10 lower (from 0.80 lower to 0.60 higher)	⊕⊕◯◯ Low	Important
**Mortality at 30 days: medications**												
1 RCT		Not serious	Not serious	Not serious	Very serious	None	1 of 18 (6)	5 of 23 (22)	OR 0.21 (0.02, 2.00)	162 fewer (from 212 fewer to 140 more) per 1000	⊕⊕◯◯ Low	Important
**Readmission rates: medications**												
1 RCT		Not serious	Not serious	Not serious	Very serious	None	0 of 11 (0)	1 of 9 (11)	OR 0.25 (0.01, 6.82)	81 fewer (from 110 fewer to 349 more) per 1000	⊕⊕◯◯ Low	Important
**Duration of hospital stay: medications**												
1 RCT		Not serious	Not serious	Not serious	Serious	None	18	23		MD 0.06 higher (from 0.56 lower to 0.67 higher)	⊕⊕⊕◯ Moderate	Important

Values in parentheses are *percentages and ^†^95 per cent confidence intervals. GRADE, Grading of Recommendations Assessment, Development and Evaluation; MD, mean difference; OR, odds ratio.

## Discussion

This systematic review of RCTs of interventions that aim to reduce postoperative sarcopenia identified seventy trials, and the vast majority had important methodological limitations; only nine trials were at low risk of allocation concealment bias. Exercise interventions were shown consistently to improve measures of sarcopenia, whether defined by muscle mass, muscle strength, timed tests or gait speed, whereas dietary interventions did not. One analysis of two trials of pharmacological interventions suggested an improvement in muscle mass attributable to the interventions. Further subgroup analyses indicated that interventions in the early and possibly late postoperative periods were most likely to have a positive effect than those undertaken before surgery. Treatment effects were independent of age. Treatment effects on muscle mass and muscle strength, but not timed tests or gait speed, were attenuated by the presence of cancer. The findings of sensitivity analyses restricted to trials at low risk of allocation concealment bias or attrition bias were comparable to the results of the primary analysis.

The review used contemporary, standardized review methods to test a prespecified hypothesis described in a prospectively registered protocol. However, it is limited by the quality and small sample size of the included studies, and the potential heterogeneity of outcome measures included in the analyses. Where possible these outcome measures were grouped according to the type of sarcopenia measure: muscle mass, muscle strength, timed test results, or gait speed (or equivalent). The results provided useful insights into a clinical problem where progress is limited by a lack of standardization and consensus definitions of outcomes.

Exercise interventions, targeted at all patient regardless of age, in the early, or possibly late, postoperative phase could reduce sarcopenia. Postoperative exercise interventions are not part of standard perioperative care, presumably owing to gaps in knowledge. On this basis, a trial of postoperative exercise interventions may be warranted. Preoperative exercise, often as part of a prehabilitation programme, is increasingly being advocated as part of enhanced recovery for people undergoing surgery. However, only two trials with small sample sizes evaluating preoperative interventions were included in the present analysis. The evaluation of treatment effects by surgical specialty was also limited by small numbers of trials in many of the prespecified subgroups. These are further knowledge gaps identified by the present review.

Evaluation of the evidence using the GRADE assessment tool identified the need to downgrade the certainty of evidence to low or very low, except for evidence presented for exercise interventions. Common reasons can be identified across all primary and secondary outcomes. First, there was a serious risk of bias across most trials. This was mainly attributable to the uncertainty around the blinding of participants, personnel and assessors across the included trials; as most of the interventions required that participants completed an exercise or nutritional programme, the lack of blinding of participants could have affected concordance and affected outcomes. Furthermore, as some assessments of muscle mass, such as the 6MWT, require volitional effort, lack of blinding could have affected the results of the assessments of such measures.

Another relevant source of bias was the large proportion of trials reporting incomplete outcomes, with high rates of participants excluded from the final analysis or lost to follow-up. This is a significant issue not only because of the volitional nature of the interventions, but also because exclusion of participants who had developed complications or required readmission to ICU may have led to significant overestimation of the benefits of intervention.

The impact of small numbers of participants on the certainty of the evidence was less severe in trials of exercise interventions, thus increasing confidence in the treatment effects of exercise programmes.

A tertiary aim of the analysis was to review the use of different methods for measurement of sarcopenia in RCTs. Muscle mass and muscle strength offer objective and highly reproducible measures of sarcopenia; however, these may miss more qualitative aspects, including changes in motivation or cognition, that may influence the outcomes of functional and semiquantitative measures such as timed tests or gait speed. Heterogeneity of effect across different measures of sarcopenia was observed in multiple analyses, but this provided limited insights into the best measurement for clinical trials. For example, in the cancer subgroup analysis, there was no treatment effect on muscle mass or strength for people with cancer; however, for measures of gait speed, a benefit was observed for patients with cancer.The reasons for this are unclear. One approach to establishing the value of different measures of sarcopenia is to look for associations with clinically important outcomes. This was not possible in the present analysis owing to the limited number of trials that reported any clinical outcomes. There was general consistency of treatment effects between primary and some secondary outcomes, with agreement between treatment effects on muscle strength and mass in most analyses (primary analysis, type of treatment, timing of intervention, cancer *versus* no cancer). Discordance in other analyses was generally an issue of precision of the estimate rather than concerning the direction of the treatment effect. These were short-term assessments of well-being, however, and the association between measures of sarcopenia and long-term outcomes and quality of life is a research priority identified by this review.

Although this systematic review of RCTs indicates that exercise interventions are likely to reduce the severity of sarcopenia after surgery, this issue should be evaluated further. Other areas of uncertainty identified by this work include the need for validation of commonly used measures with respect to long-term outcomes, the role of exercise intervention in patients with cancer, and the role of preoperative exercise interventions on sarcopenia and long-term outcomes.

## Funding

This study was funded by the Leicester National Institute for Health Research Biomedical Research Centre, and the British Heart Foundation (CH/12/1/29419, AA18/3/34220).

*Disclosure.* The authors declare no conflict of interest.

## Supplementary material

Supplementary material is available at *BJS* online.

## Supplementary Material

zraa069_Supplementary_Data
