# Supplementary material for: Interventions to prevent and treat sarcopenia in a surgical population: a systematic review and meta-analysis
Source: BJS Open. 2021 Jun 24;5(3):zraa069. doi: 10.1093/bjsopen/zraa069 (PMC8226286; doi:10.1093/bjsopen/zraa069)
Supplement: zraa069_Supplementary_Data [file zraa069_supplementary_data.zip › Supplementary materials-BJS open.docx]

Supplementary Table 1- Summary of included trials, including year and country of publication, participants inclusion criteria, intervention, comparator and main outcomes.

| **First author** | **Country** | | **Number of patients** | | **Participants** | **Intervention** | **Comparator** | **Outcomes** |
| --- | --- | --- | --- | --- | --- | --- | --- | --- |
|  | |  | |  |  |  |  |  |
| Adams a 2016 (15) | Canada | | 119 | | Women undergoing adjuvant chemotherapy for breast cancer | Aerobic exercise training | Usual care | Skeletal muscle index, muscle function, quality of life, prevalence of sarcopenia and dynapenia |
| Adams b 2016 (15) | Canada | | 123 | | Women undergoing adjuvant chemotherapy for breast cancer | Resistance exercise training | Usual care | Skeletal muscle index, muscle function, quality of life, prevalence of sarcopenia and dynapenia |
| Arbane 2011 (16) | United Kingdom | | 53 | | Patients with non-small cell lung cancer | Strength and mobility training | Usual care | Exercise tolerance, muscle strength, quality of life |
| Battaglini 2007 (17) | USA | | 20 | | Patients with breast cancer | Exercise | Usual care | Muscle strength and body composition |
| Beattie 2000 (18) | United Kingdom | | 109 | | Patients undergoing gastrointestinal and vascular surgery | Nutritional supplementation | Usual care | Nutritional status, morbidity and quality of life |
| Benedetti 2019 (19) | Italy | | 99 | | Patients undergoing a hip replacement | Abductor strengthening exercises | Usual care | Leg length discrepancy, hip range of motion, WOMAC Harris Hip score, abductor strength, 10- meter walk test, patient satisfaction using the 12-Item Short-Form Health survey) |
| Botella-Carretero 2010 (20) | Spain | | 60 | | Geriatric patients undergoing surgery for hip fracture | Nutritional supplementation | Usual care | Primary outcome: serum albumin, pre-albumin and retinol binding globulin. Secondary outcomes: changes in weight, BMI, mid-brachial circumference, tricipital fold, tolerance to the prescribed ONS, length of hospital stay, postoperative complications and the time from surgery to the start of mobilisation |
| Bruce 2003 (21) | Australia | | 109 | | Female geriatric patients undergoing surgery for hip fracture | Nutritional supplementation | Usual care | Weight loss, morality rate, discharge destination, activities of daily living, length of hospital stay |
| Bulger 2014 (22) | USA | | 41 | | Critically ill surgical and trauma patient in ICU | Oxandrolone | Placebo | The primary outcome was time on the ventilator, as a marker of restoration of lean muscle mass and improved respiratory function. Secondary outcomes included nutritional parameters, lean muscle mass as measured by bioelectrical impedance, infectious complications, development of acute respiratory distress syndrome (ARDS) or multiple organ dysfunction syndrome (MODS), rate of reintubation, length of ICU and hospital stay and mortality. |
| Busch 2012 (23) | Germany | | 121 | | Elderly patients undergoing coronary artery bypass grafting (CABG) | Intensive functional exercise training | Cardiac rehabilitation (CR) alone | Functional capacity assessed with a variety of measures (6MWT, TUG and a maximal isometric strength test); health-related quality of life (assessed through the MacNew questionnaire) |
| Delitto 1988 (24) | USA | | 20 | | Adults undergoing anterior cruciate ligament (ACL) reconstruction | Electrostimulation | Usual care | Isometric extension and flexion torque with knee at 65 degrees |
| Do 2014 (25) | United Kingdom | | 40 | | Patients who have undergone surgery for gynaecological cancers | Complex rehabilitation programme (CR) and complex decongestive therapy (CDT) (CRCDT) | Complex decongestive therapy (CDT) alone | Leg volume, bioimpedance, GCLQ (Korean version of the Gynaecological Cancer Lymphoedema Questionnaire) score, muscular strength, 30s chair stand test and QoL questionnaire |
| Dreyer 2013 (26) | USA | | 28 | | Older adults undergoing total knee arthroplasty (TKA) | Essential amino-acid (EAA) supplementation | Placebo | Changes in muscle volume and functional mobility |
| Fitzgerald 2003 (27) | USA | | 43 | | Patients undergoing ACL reconstruction | Modified neuromuscular electrostimulation (NMES) protocol | Standard rehabilitation protocol | Quadricep muscle strength and self-reported measures of knee function |
| Flodin a 2015 (28) | Sweden | | 38 | | Patients suffering from hip fracture | Protein and energy supplementation with risedronate | Vitamin D and calcium supplementation alone | Primary outcomes: changes in body composition, hand-grip strength and health-related quality of life (HRQoL). |
| Flodin b 2015 (28) | Sweden | | 41 | | Patients suffering from hip fracture | Risedronate alone | Vitamin D and calcium supplementation alone | Primary outcomes: changes in body composition, hand-grip strength and health-related quality of life (HRQoL). |
| Friedmann-Bette 2018 (29) | Germany | | 37 | | Athletes who had sustained an ACL rupture | Computer-guided leg press training with eccentric overload (CON/ECC+) | Usual leg press training (CON/ECC) | Quadricep muscle cross-sectional area and strength, number of satellite cells, evidence of myogenic activation |
| Gerber 2007 (30) | USA | | 40 | | Moderately active adults undergoing ACL reconstruction | Eccentric rehabilitation exercise programme | Usual care | The volume (in cubic centimeters) and peak cross-sectional area (in square centimeters), of the thigh muscles. |
| Ghroubi 2013 (31) | Tunisia | | 32 | | Patients undergoing CABG | Low intensity dynamic resistance training (ST) | Aerobic training (AT) | Muscle strength, maximal oxygen uptake (VO_2_ max), haemodynamic and anthropometric parameters, quality of life |
| Granger 2012 (32) | Australia | | 15 | | Patients undergoing lung resection for lung cancer | Exercise programme with a combination of resistance and aerobic exercise | Usual care | Primary outcomes: safety and feasibility of the exercise programme. Secondary outcomes: changes in functional capacity, functional mobility and HRQoL. |
| Grapar Zargi 2016 (33) | Slovenia | | 26 | | Patients undergoing ACL reconstruction | Low load resistance training with blood flow obstruction | Sham procedure | Quadricep femoris volume |
| Günes 2018 (34) | Turkey | | 68 | | Patients undergoing laparoscopic sleeve gastrectomy (LSG) | Protein supplementation (1.2g/kg/day) | Usual diet | Muscle volume of erector spinae and gastrocnemius by ultrasound scan (USS), bio-impedance analysis and SF-36 |
| Hasegawa 2011  (35) | Japan | | 20 | | Patients undergoing ACL reconstruction | Electrostimulation (EMS) | Usual care | Cross-sectional area of the muscles of the operated leg, strength and peak torque of the operated leg, Lyshom score |
| Hedström 2004 (36) | Sweden | | 20 | | Patients operated on for hip fracture | Recombinant human growth hormone (GH) | Placebo | Serum IGF-1 levels, lean body mass |
| Hermann 2016 (37) | Denmark | | 80 | | All patients over 50 scheduled for total hip arthroplasty (THA) | Explosive-type resistance training (RT) | Usual pre-operative care | Primary outcomes: Hip disability and Osteoarthritis Outcome Score (HOOS) function. Secondary outcomes: changes on self-evaluated pain/symptoms/sports and recreational function and hip related quality of life, leg extension power |
| Houborg 2006 (38) | Denmark | | 119 | | Patients aged 60 or over undergoing abdominal surgery | Exercise programme aimed at increasing strength | Rehabilitation programme aimed at improving function in activity of daily living. | Change in isometric knee extension strength and fatigue 30 day post-operatively |
| Invernizzi 2019 (39) | Italy | | 32 | | Osteoporotic hip fracture patients | Dietary amminoacid supplementation with exercise programme | Exercise programme with dietetics input but no supplementation. | Primary outcome: appendicular muscle strength measured by hand grip strength test (HGS), physical performance using the Timed Up and Go test (TUG), the level of assistance, measured by the Iowa Level of Assistance scale (ILOA). Secondary outcomes were nutritional assessments with evaluation of daily caloric intake and daily protein intake and the health-related quality of life (HRQoL) using the 12-Item Short Form Health Survey (SF-12) determining both Physical (PCS) and Mental Health Composite Scores (MCS). |
| Iversen 2015 (40) | Norway | | 24 | | Athletes who had sustained an ACL rupture | Low load resistance training with blood flow obstruction | Exercise alone | Quadricep anatomical cross-section area (ACSA). |
| Jensen a 1997 (41) | Denmark | | 32 | | Elective patients undergoing colorectal surgery under the age of 75 | Nutritional intervention: dietetic advice and high protein or high protein/high energy supplement | Usual care | Changes in nutritional intake and body composition |
| Jensen b 1997 (41) | Denmark | | 20 | | Elective patients undergoing colorectal surgery over the age of 75 | Nutritional intervention: dietetic advice and high protein or high protein/high energy supplement | Usual care | Changes in nutritional intake and body composition |
| Jensen c 1997 (41) | Denmark | | 21 | | Patients undergoing emergency abdominal surgery under the age of 75 | Nutritional intervention: dietetic advice and high protein or high protein/high energy supplement | Usual care | Changes in nutritional intake and body composition |
| Jensen d 1997 (41) | Denmark | | 14 | | Patients undergoing emergency abdominal surgery over the age of 75 | Nutritional intervention: dietetic advice and high protein or high protein/high energy supplement | Usual care | Changes in nutritional intake and body composition |
| Karelis 2016 (42) | Canada | | 24 | | Kidney transplants recipients | Resistance training | Sedentary lifestyle | Insulin resistance; glucose tolerance test; body composition, health-related quality of life |
| Lambert 2019 (43) | USA | | 14 | | Healthy young adults | Blood-flow restricted exercise | Exercise alone | Bone Mineral Density, bone mass, lean muscle mass |
| Lattanzi 2019 (44) | Italy | | 22 | | Males undergoing liver transplant | Beta-hydroxy-beta-methylbutyrate (HMB) | Placebo | Muscle function and muscle mass, safety profile, long term effect of HMB |
| Liao 2019  (45) | Taiwan | | 60 | | Women undergoing total knee replacement | Resistance elastic training (RET) | Usual care | Walk capability, balance, muscle strength, Western Ontario and McMaster Universities Osteoarthritis Index |
| Lima 2019  (46) | Brazil | | 12 | | Kidney transplants recipients | Supervised exercise programme | Usual care | Changes in body composition, muscular strength, aerobic, and renal function. |
| Macchi 2007  (47) | Italy | | 300 | | Cardiac surgery patients | Early rehabilitation programme | Usual care | Impact on long term outcomes including physical functioning. Safety of early rehabilitation programme |
| Maiorana 1997 (48) | Italy | | 26 | | Male cardiac surgery patients | Circuit weight training (CWT) | Usual care | Muscular strength, peak oxygen consumption, and myocardial oxygen demand |
| Malafarina 2017  (49) | Spain | | 107 | | Patients suffering from hip fracture | Beta-hydroxy-beta-methylbutyrate (HMB) | Usual diet | Muscle mass, nutritional markers (BMI, proteins) |
| Morano 2013 (50) | Spain | | 24 | | Patient undergoing lung cancer resection | Pulmonary rehabilitation (PR) | Usual care | Functional parameters (pre-operatively) and pulmonary complications (post-op) |
| Nishikazi 2015  (51) | Japan | | 23 | | Patients undergoing total knee replacement for osteoarthritis | Beta-hydroxy-beta-methylbutyrate, arginine and glutamine supplementation | Placebo | Quadriceps muscle strength |
| Noyes 1987 (52) | USA | | 18 | | Patients undergoing ACL repair | Continuous passive mobilisation of the knee joint | Usual care | Knee range of movement, thigh circumference, |
| Ohta 2003  (53) | Japan | | 44 | | Patients undergoing ACL repair | Oxygen-restricted resistance exercise | Resistance exercise alone | Quadricep strength and amount of muscle mass |
| Oppert a 2018  (54) | France | | 42 | | Patients undergoing Roux-en-Y gastric bypass | Whey protein supplementation | Usual care | Lean body mass and strength |
| Oppert b 2018 (54) | France | | 34 | | Patients undergoing Roux-en-Y gastric bypass | Whey protein supplementation and resistance exercise | Usual care | Lean body mass and strength |
| Paternostro-Sluga a 1998 (55) | Austria | | 25 | | Patients undergoing ACL reconstruction | Neuromuscular electrostimulation | Exercise alone | Isometric and isokinetic knee extensor and flexor torque |
| Paternostro-Sluga b 1998 (55) | Austria | | 22 | | Patients undergoing ACL reconstruction | Subcutaneous electrical stimulation | Exercise alone | Isometric and isokinetic knee extensor and flexor torque |
| Ritch 2019  (56) | USA | | 61 | | Patients undergoing radical cystectomy | Oral nutrition supplement with fatty acids and HMB | Multivitamin | 30-day free hospital days, changes in total and lean body mass |
| Ross 1999  (57) | USA | | 20 | | Patients undergoing ACL repair | Neuromuscular electrostimulation (NMES) | Closed kinetic chain exercises (CKCh) | Anterior tibiofemoral joint laxity and the following standard closed kinetic chain #performance activities: (1) unilateral squat, (2) 0.10m lateral step-up test and (3) anterior reach test. |
| Salvarani 2003 (58) | Italy | | 20 | | Patients undergoing ACL repair | Mechanical vibration | Usual care | Lower limb muscle strength during isometric contraction |
| Savastano 2009  (59) | Italy | | 23 | | Patients undergoing laparoscopic-assisted gastric banding (LASGB) | Growth hormone (GH) | Usual care | Primary outcomes: percentage of excess body weight loss, decrease of fat mass and lean body mass. Secondary outcomes: BMI, waist circumference, appendicular skeletal muscle mass and glucose tolerance. |
| Schürch 1998 (60) | Switzerland | | 82 | | Hip fracture patients | High protein supplement | Isocaloric placebo | Bone mineral density and IGF-1 levels |
| Shaarani 2013 (61) | Ireland | | 25 | | Patients undergoing ACL repair (ACLR) | Prehabilitation programme | Usual care | Isokinetic strength, dynamic function, muscle cross-sectional area, self-reported assessment and molecular effects. |
| Singh 2012 (62) | Australia | | 124 | | Elderly patients following admission for a fracture of neck of femur | Geriatrician-supervised high intensity weight-lifting exercise and targeted treatment of balance, osteoporosis, nutrition, vitamin D/calcium, depression, cognition, vision, home safety, polypharmacy, hip protector, efficacy and social support | Usual care | Functional independence: mortality, nursing home admission, basic and instrumental activities of daily living and assistive device utilisation |
| Sisk 1987 (63) | USA | | 22 | | Patients undergoing ACL repair | Neuromuscular electrostimulation (ES) | Isometric exercise | Isometric quadriceps strength |
| Snyder-Mackler 1991 (64) | USA | | 10 | | Patients undergoing ACL reconstruction | Electrostimulation and volitional exercise | Exercise alone | Gait analysis, quadriceps and hamstring torque |
| Stigt 2013 (65) | Netherlands | | 49 | | Patients with resectable lung cancer undergoing thoracotomy | Pulmonary rehabilitation | Usual care | Primary outcomes: changes in quality of life measures. Secondary outcomes: changes in exercise tolerance and pain sensation. Feasibility of programme in patients undergoing chemotherapy. |
| Suetta 2a 2004 (66) | Denmark | | 19 | | 36 elderly patients scheduled for unilateral hip replacement | Unilateral resistance training and standard rehabilitation | Usual care | Hospital length of stay, quadricep muscle cross-sectional area and maximal quadricep strength |
| Suetta 2b 2004 (66) | Denmark | | 18 | | 36 elderly patients scheduled for unilateral hip replacement | Unilateral electrical stimulation of the quadriceps muscle and standard rehabilitation | Usual care | Hospital length of stay, quadricep muscle cross-sectional area and maximal quadricep strength |
| Takarada 2000 (67) | Japan | | 16 | | Healthy adults undergoing ACL reconstruction | Occlusive stimuli | Sham procedure | Knee extensor muscle mass and cross-sectional area (CSA) |
| Tengstrand a 2004  (68) | Sweden | | 40 | | Women over 70 years of age who suffered a hip fracture suitable for surgical fixation | Protein supplementation | Usual care | Nutritional and functional status, health-related quality of life |
| Tengstrand b 2004  (68) | Sweden | | 39 | | Women over 70 years of age who suffered a hip fracture suitable for surgical fixation | Protein supplementation and nandrolone decanoate | Usual care | Nutritional and functional status, health-related quality of life |
| Tsukagoshi a 2014  (69) | Japan | | 33 | | Women who had undergone hip arthroplasty for osteoarthritis | Weight bearing exercise | Usual care | Functional performance, disease-specific functional outcome measure, muscle strength and muscle thickness |
| Tsukagoshi b 2014  (69) | Japan | | 32 | | Women who had undergone hip arthroplasty for osteoarthritis | Non weight bearing exercise | Usual care | Functional performance, disease-specific functional outcome measure, muscle strength and muscle thickness |
| Van Meerbeeck a 2013  (70) | Belgium | | 36 | | Patients with radically treated lung cancer | Conventional resistance training | Usual care | Primary outcome: differences in the 6MWT. Secondary outcomes: maximal exercise capacity (Wmax), muscle strength (Quadriceps Force (QF)) and QoL (physical functioning (PF), fatigue (F), pain (P) and dyspnea D)) |
| Van Meerbeeck b 2013  (70) | Belgium | | 34 | | Patients with radically treated lung cancer | Whole body vibration | Usual care | Primary outcome: differences in the 6MWT. Secondary outcomes: maximal exercise capacity (Wmax), muscle strength (Quadriceps Force (QF)) and QoL (physical functioning (PF), fatigue (F), pain (P) and dyspnea D)) |
| Wigerstad-Lossing 1988  (71) | Sweden | | 20 | | Patients undergoing ACL repair | Electrostimulation and isometric exercise | Exercise alone | Quadricep isometric strength, quadriceps cross-sectional area, muscle fiber relative area and activity of citrate synthase and triphosphate dehydrogenase |
| Wu (72) | USA | | 13 | | Men undergoing surgery for ruptured ACL | Testosterone 200 mg/week intramuscular | Placebo | Primary outcome: change in total lean body mass. Secondary outcomes: muscle strength and Knee Injury and Osteoarthritis Score (KOOS) |
| Ximenes (73) | Brazil | | 34 | | Patients undergoing CABG | Resistance exercise | Usual care | Functional capacity and pulmonary function |

Supplementary Table 2- Summary of treatment effects for primary outcomes with subgroup analysis for the pre-specified source of heterogeneity (type of surgical intervention, age, timing of intervention in relation to surgery and cancer status).

| **Subgroup analysis: type of surgery** | | | | | |
| --- | --- | --- | --- | --- | --- |
| **Subgroup** | **Studies** | **Participants** | **Treatment effect [95% C.I.]** | **p value** | **Heterogeneity (I^2^)**  **(p value)** |
| **Quantitative measures of muscle mass** | | | | | |
| Orthopaedic surgery | 16 | 453 | SMD 0.57 [0.32, 0.83] | <0.0001 | 38%  (p=0.06) |
| Breast surgery | 3 | 262 | SMD 0.30 [-0.23, 0.84] | 0.26 | 70%  (p=0.04) |
| Urology | 1 | 61 | SMD -0.42 [-0.93, 0.09] | 0.1 | Not estimable |
| Cardiothoracic surgery | 0 | 0 | Not estimable | Not estimable | Not estimable |
| General surgery | 0 | 0 | Not estimable | Not estimable | Not estimable |
| Bariatric surgery | 1 | 23 | SMD 1.12 [0.23, 2.02] | 0.01 | Not estimable |
| Transplant surgery | 3 | 53 | SMD 0.34 [-0.21, 0.90] | 0.22 | 0%  (p=0.43) |
| Gynaecological surgery | 0 | 0 | Not estimable | Not estimable | Not estimable |
| Trauma | 0 | 0 | Not estimable | Not estimable | Not estimable |
| Heterogeneity between subgroups (I^2^) = 72.4%, p=0.006 | | | | | |
| **Quantitative measures of muscle strength** | | | | | |
| Orthopaedic surgery | 12 | 472 | SMD 0.49 [0.27, 0.72] | <0.0001 | 22%  (p=0.22) |
| Breast surgery | 0 | 0 | Not estimable | Not estimable | Not estimable |
| Urology | 0 | 0 | Not estimable | Not estimable | Not estimable |
| Cardiothoracic surgery | 3 | 185 | 0.42 [0.13, 0.72] | 0.005 | 0%  (p=0.48) |
| General surgery | 0 | 0 | Not estimable | Not estimable | Not estimable |
| Bariatric surgery | 0 | 0 | Not estimable | Not estimable | Not estimable |
| Transplant surgery | 2 | 33 | SMD 0.57 [-0.89, 2.03] | 0.45 | 71%  (p=0.06) |
| Gynaecological surgery | 1 | 40 | SMD 0.53 [-0.10, 1.16] | 0.1 | Not estimable |
| Trauma | 0 | 0 | Not estimable | Not estimable | Not estimable |
| Heterogeneity between subgroups (I^2^) = 0%, p=0.98 | | | | | |
| **Timed tests** | | | | | |
| Orthopaedic surgery | 6 | 258 | SMD -0.84 [-1.25, -0.44] | <0.0001 | 49%  (p=0.08) |
| Breast surgery | 0 | 0 | Not estimable | Not estimable | Not estimable |
| Urology | 0 | 0 | Not estimable | Not estimable | Not estimable |
| Cardiothoracic surgery | 2 | 153 | SMD -0.64 [-1.62, 0.34] | 0.2 | 55%  (p=0.14) |
| General surgery | 0 | 0 | Not estimable | Not estimable | Not estimable |
| Bariatric surgery | 0 | 0 | Not estimable | Not estimable | Not estimable |
| Transplant surgery | 1 | 21 | SMD -0.76 [-1.66, 0.14] | 0.1 | Not estimable |
| Gynaecological surgery | 0 | 0 | Not estimable | Not estimable | Not estimable |
| Trauma | 0 | 0 | Not estimable | Not estimable | Not estimable |
| Heterogeneity between subgroups (I^2^) = 0%, p=0.93 | | | | | |
| **Repetition-based tests** | | | | | |
| Orthopaedic surgery | 3 | 171 | SMD 0.14 [-0.16, 0.45] | 0.35 | 0%  (p=0.97) |
| Breast surgery | 0 | 0 | Not estimable | Not estimable | Not estimable |
| Urology | 0 | 0 | Not estimable | Not estimable | Not estimable |
| Cardiothoracic surgery | 6 | 420 | SMD 0.32 [-0.10, 0.75] | 0.14 | 55%  (p=0.05) |
| General surgery | 0 | 0 | Not estimable | Not estimable | Not estimable |
| Bariatric surgery | 0 | 0 | Not estimable | Not estimable | Not estimable |
| Transplant surgery | 0 | 0 | Not estimable | Not estimable | Not estimable |
| Gynaecological surgery | 1 | 40 | SMD 1.13 [0.46, 1.80] | 0.001 | Not estimable |
| Trauma | 0 | 0 | Not estimable | Not estimable | Not estimable |
| Heterogeneity between subgroups (I^2^) = 70.7%, p=0.03 | | | | | |
| **Subgroup analysis: age** | | | | | |
| **Subgroup** | **Studies** | **Participants** | **Treatment effect [95% C.I.]** | **p value** | **Heterogeneity (I^2^)**  **(p value)** |
| **Quantitative measures of muscle mass** | | | | | |
| Under 65 | 17 | 573 | SMD 0.63 [0.34, 0.92] | <0.0001 | 9%  (p=0.35) |
| Over 65 | 6 | 218 | SMD 0.35 [0.08, 0.63] | 0.01 | 68%  (p=0.01) |
| Heterogeneity between subgroups (I^2^) = 0%, p=0.17 | | | | | |
| **Quantitative measures of muscle strength** | | | | | |
| Under 65 | 9 | 319 | SMD 0.45 [0.16, 0.74] | 0.003 | 33%  (p=0.15) |
| Over 65 | 6 | 339 | SMD 0.43 [0.21, 0.66] | 0.002 | 5%  (p=0.39) |
| Heterogeneity between subgroups (I^2^) = 0%, p=0.94 | | | | | |
| **Timed tests** | | | | | |
| Under 65 | 4 | 189 | SMD -0.70 [-1.02, -0.37] | <0.0001 | 9%  (p=0.35) |
| Over 65 | 5 | 243 | SMD -0.77 [-1.38, -0.15] | 0.01 | 68%  (p=0.01) |
| Heterogeneity between subgroups (I^2^) = 0%, p=0.84 | | | | | |
| **Repetition-bases tests** | | | | | |
| Under 65 | 4 | 128 | SMD 0.39 [-0.21, 0.98] | 0.2 | 63%  (p=0.05) |
| Over 65 | 5 | 203 | SMD 0.51 [-0.12, 1.14] | 0.11 | 56%  (p=0.06) |
| Heterogeneity between subgroups (I^2^) = 0%, p=0.78 | | | | | |
| **Subgroup analysis: timing of interventions** | | | | | |
| **Subgroup** | **Studies** | **Participants** | **Treatment effect [95% C.I.]** | **p value** | **Heterogeneity (I^2^)**  **(p value)** |
| **Quantitative measures of muscle mass** | | | | | |
| Pre-operative | 2 | 40 | SMD 1.27 [-0.20, 2.73] | 0.09 | 76%  (p=0.09) |
| Peri-operative | 4 | 125 | SMD -0.07 [-0.46, 0.33] | 0.74 | 13%  (p=0.33) |
| Early post-operative | 11 | 308 | SMD 0.71 [0.35, 1.07] | <0.0001 | 49%  (p=0.03) |
| Late post-operative | 7 | 376 | SMD 0.20 [-0.01, 0.42] | 0.2 | 0%  (p=0.55) |
| Heterogeneity between subgroups (I^2^) = 72.3%, p=0.01 | | | | | |
| **Quantitative measures of muscle strength** | | | | | |
| Pre-operative | 1 | 80 | SMD 0.83 [0.37, 1.28] | 0.0004 | Not estimable |
| Peri-operative | 0 | 0 | Not estimable | Not estimable | Not estimable |
| Early post-operative | 11 | 453 | SMD 0.51 [0.31, 0.70] | <0.00001 | 3%  (p=0.42) |
| Late post-operative | 4 | 118 | SMD 0.43 [-0.04, 0.91] | 0.07 | 34%  (p=0.21) |
| Heterogeneity between subgroups (I^2^) = 0%, p=0.41 | | | | | |
| **Times tests** | | | | | |
| Pre-operative | 0 | 0 | Not estimable | Not estimable | Not estimable |
| Peri-operative | 0 | 0 | Not estimable | Not estimable | Not estimable |
| Early post-operative | 7 | 367 | SMD -0.70 [-1.10, -0.30] | 0.005 | 59%  (p=0.02) |
| Late post-operative | 2 | 65 | SMD -0.64 [-1.17, -0.11] | 0.02 | 0%  (p=0.51) |
| Heterogeneity between subgroups (I^2^) = 0%, p=0.02 | | | | | |
| **Repetition-based tests** | | | | | |
| Pre-operative | 1 | 24 | SMD 1.39 [0.48, 2.30] | 0.003 | Not estimable |
| Peri-operative | 0 | 0 | Not estimable | Not estimable | Not estimable |
| Early post-operative | 7 | 536 | SMD 0.21 [-0.03, 0.46] | 0.09 | 23%  (p=0.25) |
| Late post-operative | 2 | 72 | SMD 0.44 [-0.92, 1.80] | 0.53 | 87%  (p=0.005) |
| Heterogeneity between subgroups (I^2^) = 67%, p=0.05 | | | | | |
| **Subgroup analysis: cancer status** | | | | | |
| **Subgroup** | **Studies** | **Participants** | **Effect estimate** | **p value** | **Heterogeneity (I^2^)**  **(p value)** |
| **Quantitative measures of muscle mass** | | | | | |
| Cancer | 5 | 344 | SMD 0.09 [-0.31, 0.49] | 0.66 | 62%  (p=0.03) |
| No cancer | 17 | 472 | SMD 0.58 [0.33, 0.83] | <0.00001 | 36%  (p=0.07) |
| Heterogeneity between subgroups (I^2^) = 76%, p=0.04 | | | | | |
| **Quantitative measures of muscle strength** | | | | | |
| Cancer | 3 | 91 | SMD 0.46 [-0.01, 0.92] | 0.05 | 16%  (p=0.3) |
| No cancer | 18 | 702 | SMD 0.49 [0.34, 0.65] | <0.00001 | 4%  (p=0.41) |
| Heterogeneity between subgroups (I^2^) = 0%, p=0.89 | | | | | |
| **Timed tests** | | | | | |
| Cancer | 2 | 32 | SMD -0.95 [-1.71, -0.19] | 0.01 | 0%  (p=0.45) |
| No cancer | 7 | 400 | SMD -0.72 [-1.09, -0.36] | 0.0001 | 59%  (p=0.02) |
| Heterogeneity between subgroups (I^2^) = 0%, p=0.6 | | | | | |
| **Repetition-based tests** | | | | | |
| Cancer | 5 | 128 | SMD 0.80 [0.22, 1.39] | 0.007 | 52%  (p=0.08) |
| No cancer | 5 | 503 | SMD 0.12 [-0.05, 0.30] | 0.17 | 0%  (p=0.86) |
| Heterogeneity between subgroups (I^2^) = 79.1%, p=0.03 | | | | | |

Supplementary Table 3- Summary of treatment effects for primary outcomes with subgroup analysis for the pre-specified source of heterogeneity (type of surgical intervention, age, timing of intervention in relation to surgery and cancer status).

| **Subgroup analysis: type of surgery** | | | | | |
| --- | --- | --- | --- | --- | --- |
| **Subgroup** | **Studies** | **Participants** | **Treatment effect [95% C.I.]** | **p value** | **Heterogeneity (I^2^)**  **(p value)** |
| **Quantitative measures of muscle mass** | | | | | |
| Orthopaedic surgery | 16 | 453 | SMD 0.57 [0.32, 0.83] | <0.0001 | 38%  (p=0.06) |
| Breast surgery | 3 | 262 | SMD 0.30 [-0.23, 0.84] | 0.26 | 70%  (p=0.04) |
| Urology | 1 | 61 | SMD -0.42 [-0.93, 0.09] | 0.1 | Not estimable |
| Cardiothoracic surgery | 0 | 0 | Not estimable | Not estimable | Not estimable |
| General surgery | 0 | 0 | Not estimable | Not estimable | Not estimable |
| Bariatric surgery | 1 | 23 | SMD 1.12 [0.23, 2.02] | 0.01 | Not estimable |
| Transplant surgery | 3 | 53 | SMD 0.34 [-0.21, 0.90] | 0.22 | 0%  (p=0.43) |
| Gynaecological surgery | 0 | 0 | Not estimable | Not estimable | Not estimable |
| Trauma | 0 | 0 | Not estimable | Not estimable | Not estimable |
| Heterogeneity between subgroups (I^2^) = 72.4%, p=0.006 | | | | | |
| **Quantitative measures of muscle strength** | | | | | |
| Orthopaedic surgery | 12 | 472 | SMD 0.49 [0.27, 0.72] | <0.0001 | 22%  (p=0.22) |
| Breast surgery | 0 | 0 | Not estimable | Not estimable | Not estimable |
| Urology | 0 | 0 | Not estimable | Not estimable | Not estimable |
| Cardiothoracic surgery | 3 | 185 | 0.42 [0.13, 0.72] | 0.005 | 0%  (p=0.48) |
| General surgery | 0 | 0 | Not estimable | Not estimable | Not estimable |
| Bariatric surgery | 0 | 0 | Not estimable | Not estimable | Not estimable |
| Transplant surgery | 2 | 33 | SMD 0.57 [-0.89, 2.03] | 0.45 | 71%  (p=0.06) |
| Gynaecological surgery | 1 | 40 | SMD 0.53 [-0.10, 1.16] | 0.1 | Not estimable |
| Trauma | 0 | 0 | Not estimable | Not estimable | Not estimable |
| Heterogeneity between subgroups (I^2^) = 0%, p=0.98 | | | | | |
| **Timed tests** | | | | | |
| Orthopaedic surgery | 6 | 258 | SMD -0.84 [-1.25, -0.44] | <0.0001 | 49%  (p=0.08) |
| Breast surgery | 0 | 0 | Not estimable | Not estimable | Not estimable |
| Urology | 0 | 0 | Not estimable | Not estimable | Not estimable |
| Cardiothoracic surgery | 2 | 153 | SMD -0.64 [-1.62, 0.34] | 0.2 | 55%  (p=0.14) |
| General surgery | 0 | 0 | Not estimable | Not estimable | Not estimable |
| Bariatric surgery | 0 | 0 | Not estimable | Not estimable | Not estimable |
| Transplant surgery | 1 | 21 | SMD -0.76 [-1.66, 0.14] | 0.1 | Not estimable |
| Gynaecological surgery | 0 | 0 | Not estimable | Not estimable | Not estimable |
| Trauma | 0 | 0 | Not estimable | Not estimable | Not estimable |
| Heterogeneity between subgroups (I^2^) = 0%, p=0.93 | | | | | |
| **Repetition-based tests** | | | | | |
| Orthopaedic surgery | 3 | 171 | SMD 0.14 [-0.16, 0.45] | 0.35 | 0%  (p=0.97) |
| Breast surgery | 0 | 0 | Not estimable | Not estimable | Not estimable |
| Urology | 0 | 0 | Not estimable | Not estimable | Not estimable |
| Cardiothoracic surgery | 6 | 420 | SMD 0.32 [-0.10, 0.75] | 0.14 | 55%  (p=0.05) |
| General surgery | 0 | 0 | Not estimable | Not estimable | Not estimable |
| Bariatric surgery | 0 | 0 | Not estimable | Not estimable | Not estimable |
| Transplant surgery | 0 | 0 | Not estimable | Not estimable | Not estimable |
| Gynaecological surgery | 1 | 40 | SMD 1.13 [0.46, 1.80] | 0.001 | Not estimable |
| Trauma | 0 | 0 | Not estimable | Not estimable | Not estimable |
| Heterogeneity between subgroups (I^2^) = 70.7%, p=0.03 | | | | | |
| **Subgroup analysis: age** | | | | | |
| **Subgroup** | **Studies** | **Participants** | **Treatment effect [95% C.I.]** | **p value** | **Heterogeneity (I^2^)**  **(p value)** |
| **Quantitative measures of muscle mass** | | | | | |
| Under 65 | 17 | 573 | SMD 0.63 [0.34, 0.92] | <0.0001 | 9%  (p=0.35) |
| Over 65 | 6 | 218 | SMD 0.35 [0.08, 0.63] | 0.01 | 68%  (p=0.01) |
| Heterogeneity between subgroups (I^2^) = 0%, p=0.17 | | | | | |
| **Quantitative measures of muscle strength** | | | | | |
| Under 65 | 9 | 319 | SMD 0.45 [0.16, 0.74] | 0.003 | 33%  (p=0.15) |
| Over 65 | 6 | 339 | SMD 0.43 [0.21, 0.66] | 0.002 | 5%  (p=0.39) |
| Heterogeneity between subgroups (I^2^) = 0%, p=0.94 | | | | | |
| **Timed tests** | | | | | |
| Under 65 | 4 | 189 | SMD -0.70 [-1.02, -0.37] | <0.0001 | 9%  (p=0.35) |
| Over 65 | 5 | 243 | SMD -0.77 [-1.38, -0.15] | 0.01 | 68%  (p=0.01) |
| Heterogeneity between subgroups (I^2^) = 0%, p=0.84 | | | | | |
| **Repetition-bases tests** | | | | | |
| Under 65 | 4 | 128 | SMD 0.39 [-0.21, 0.98] | 0.2 | 63%  (p=0.05) |
| Over 65 | 5 | 203 | SMD 0.51 [-0.12, 1.14] | 0.11 | 56%  (p=0.06) |
| Heterogeneity between subgroups (I^2^) = 0%, p=0.78 | | | | | |
| **Subgroup analysis: timing of interventions** | | | | | |
| **Subgroup** | **Studies** | **Participants** | **Treatment effect [95% C.I.]** | **p value** | **Heterogeneity (I^2^)**  **(p value)** |
| **Quantitative measures of muscle mass** | | | | | |
| Pre-operative | 2 | 40 | SMD 1.27 [-0.20, 2.73] | 0.09 | 76%  (p=0.09) |
| Peri-operative | 4 | 125 | SMD -0.07 [-0.46, 0.33] | 0.74 | 13%  (p=0.33) |
| Early post-operative | 11 | 308 | SMD 0.71 [0.35, 1.07] | <0.0001 | 49%  (p=0.03) |
| Late post-operative | 7 | 376 | SMD 0.20 [-0.01, 0.42] | 0.2 | 0%  (p=0.55) |
| Heterogeneity between subgroups (I^2^) = 72.3%, p=0.01 | | | | | |
| **Quantitative measures of muscle strength** | | | | | |
| Pre-operative | 1 | 80 | SMD 0.83 [0.37, 1.28] | 0.0004 | Not estimable |
| Peri-operative | 0 | 0 | Not estimable | Not estimable | Not estimable |
| Early post-operative | 11 | 453 | SMD 0.51 [0.31, 0.70] | <0.00001 | 3%  (p=0.42) |
| Late post-operative | 4 | 118 | SMD 0.43 [-0.04, 0.91] | 0.07 | 34%  (p=0.21) |
| Heterogeneity between subgroups (I^2^) = 0%, p=0.41 | | | | | |
| **Times tests** | | | | | |
| Pre-operative | 0 | 0 | Not estimable | Not estimable | Not estimable |
| Peri-operative | 0 | 0 | Not estimable | Not estimable | Not estimable |
| Early post-operative | 7 | 367 | SMD -0.70 [-1.10, -0.30] | 0.005 | 59%  (p=0.02) |
| Late post-operative | 2 | 65 | SMD -0.64 [-1.17, -0.11] | 0.02 | 0%  (p=0.51) |
| Heterogeneity between subgroups (I^2^) = 0%, p=0.02 | | | | | |
| **Repetition-based tests** | | | | | |
| Pre-operative | 1 | 24 | SMD 1.39 [0.48, 2.30] | 0.003 | Not estimable |
| Peri-operative | 0 | 0 | Not estimable | Not estimable | Not estimable |
| Early post-operative | 7 | 536 | SMD 0.21 [-0.03, 0.46] | 0.09 | 23%  (p=0.25) |
| Late post-operative | 2 | 72 | SMD 0.44 [-0.92, 1.80] | 0.53 | 87%  (p=0.005) |
| Heterogeneity between subgroups (I^2^) = 67%, p=0.05 | | | | | |
| **Subgroup analysis: cancer status** | | | | | |
| **Subgroup** | **Studies** | **Participants** | **Effect estimate** | **p value** | **Heterogeneity (I^2^)**  **(p value)** |
| **Quantitative measures of muscle mass** | | | | | |
| Cancer | 5 | 344 | SMD 0.09 [-0.31, 0.49] | 0.66 | 62%  (p=0.03) |
| No cancer | 17 | 472 | SMD 0.58 [0.33, 0.83] | <0.00001 | 36%  (p=0.07) |
| Heterogeneity between subgroups (I^2^) = 76%, p=0.04 | | | | | |
| **Quantitative measures of muscle strength** | | | | | |
| Cancer | 3 | 91 | SMD 0.46 [-0.01, 0.92] | 0.05 | 16%  (p=0.3) |
| No cancer | 18 | 702 | SMD 0.49 [0.34, 0.65] | <0.00001 | 4%  (p=0.41) |
| Heterogeneity between subgroups (I^2^) = 0%, p=0.89 | | | | | |
| **Timed tests** | | | | | |
| Cancer | 2 | 32 | SMD -0.95 [-1.71, -0.19] | 0.01 | 0%  (p=0.45) |
| No cancer | 7 | 400 | SMD -0.72 [-1.09, -0.36] | 0.0001 | 59%  (p=0.02) |
| Heterogeneity between subgroups (I^2^) = 0%, p=0.6 | | | | | |
| **Repetition-based tests** | | | | | |
| Cancer | 5 | 128 | SMD 0.80 [0.22, 1.39] | 0.007 | 52%  (p=0.08) |
| No cancer | 5 | 503 | SMD 0.12 [-0.05, 0.30] | 0.17 | 0%  (p=0.86) |
| Heterogeneity between subgroups (I^2^) = 79.1%, p=0.03 | | | | | |

Supplementary Table 4- Sensitivity analysis including only studies at low risk of bias due to allocation concealment and incomplete outcome reporting (attrition bias).

| **Subgroup analysis: type of surgery** | | | | | |
| --- | --- | --- | --- | --- | --- |
| **Subgroup** | **Studies** | **Participants** | **Treatment effect [95% C.I.]** | **p value** | **Heterogeneity (I^2^)**  **(p value)** |
| **Self-reported quality of life** | | | | | |
| Orthopaedic surgery | 4 | 250 | SMD 0.34 [-0.07, 0.76] | 0.11 | 58%  (p=0.07) |
| Breast surgery | 2 | 242 | SMD 0.11 [-0.16, 0.38] | 0.42 | 0%  (p=0.6) |
| Urology | 0 | 0 | Not estimable | Not estimable | Not estimable |
| Cardiothoracic surgery | 4 | 202 | SMD 0.13 [-0.18, 0.44] | 0.4 | 11%  (p=0.34) |
| General surgery | 1 | 101 | SMD 0.49 [0.09, 0.88] | 0.02 | Not estimable |
| Bariatric surgery | 0 | 0 | Not estimable | Not estimable | Not estimable |
| Transplant surgery | 1 | 20 | SMD 0.65 [-0.25, 1.56] | 0.16 | Not estimable |
| Gynaecological surgery | 1 | 40 | SMD 0.27 [-0.36, 0.89] | 0.4 | Not estimable |
| Trauma | 0 | 0 | Not estimable | Not estimable | Not estimable |
| Heterogeneity between subgroups (I^2^) = 0%, p= 0.56 | | | | | |
| **Discharge to higher levels of care** | | | | | |
| Orthopaedic surgery | 1 | 124 | OR 0.37 [0.12, 1.11] | 0.08 | Not estimable |
| Breast surgery | 0 | 0 | Not estimable | Not estimable | Not estimable |
| Urology | 0 | 0 | Not estimable | Not estimable | Not estimable |
| Cardiothoracic surgery | 1 | 15 | OR 0.33 [0.01, 9.57] | 0.52 | Not estimable |
| General surgery | 0 | 0 | Not estimable | Not estimable | Not estimable |
| Bariatric surgery | 0 | 0 | Not estimable | Not estimable | Not estimable |
| Transplant surgery | 0 | 0 | Not estimable | Not estimable | Not estimable |
| Heterogeneity between subgroups (I^2^) = 0%, p= 0.96 | | | | | |
| **Mortality at 30 days** | | | | | |
| Orthopaedic surgery | 1 | 20 | OR 0.25 [0.01, 6.82] | 0.41 | Not estimable |
| Breast surgery | 0 | 0 | Not estimable | Not estimable | Not estimable |
| Urology | 1 | 61 | OR 0.97 [0.06, 16.19] | 0.98 | Not estimable |
| Cardiothoracic surgery | 0 | 0 | Not estimable | Not estimable | Not estimable |
| General surgery | 0 | 0 | Not estimable | Not estimable | Not estimable |
| Bariatric surgery | 0 | 0 | Not estimable | Not estimable | Not estimable |
| Transplant surgery | 0 | 0 | Not estimable | Not estimable | Not estimable |
| Gynaecological surgery | 0 | 0 | Not estimable | Not estimable | Not estimable |
| Trauma | 1 | 41 | OR 0.21 [0.02, 2.00] | 0.18 | Not estimable |
| Heterogeneity between subgroups (I^2^) = 0%, p=0.69 | | | | | |
| **Readmission rates** | | | | | |
| Orthopaedic surgery | 0 | 0 | Not estimable | Not estimable | Not estimable |
| Breast surgery | 0 | 0 | Not estimable | Not estimable | Not estimable |
| Urology | 1 | 61 | OR 0.34 [0.06, 1.94] | 0.23 | Not estimable |
| Cardiothoracic surgery | 1 | 32 | OR 0.47 [0.04, 5.73] | 0.55 | Not estimable |
| General surgery | 0 | 0 | Not estimable | Not estimable | Not estimable |
| Bariatric surgery | 0 | 0 | Not estimable | Not estimable | Not estimable |
| Transplant surgery | 0 | 0 | Not estimable | Not estimable | Not estimable |
| Heterogeneity between subgroups (I^2^) = 0%, p=0.85 | | | | | |
| **Length of hospital stay** | | | | | |
| Orthopaedic surgery | 7 | 379 | SMD -0.10 [-0.42, 0.21] | 0.53 | 50%  (p=0.06) |
| Breast surgery | 0 | 0 | Not estimable | Not estimable | Not estimable |
| Urology | 1 | 61 | SMD -0.04 [-0.55, 0.46] | 0.86 | Not estimable |
| Cardiothoracic surgery | 5 | 423 | SMD -0.84 [-1.51, -0.18] | 0.01 | 83%  (p<0.0001) |
| General surgery | 2 | 228 | SMD -0.08 [-0.34, 0.18] | 0.54 | 0%  (p=0.52) |
| Bariatric surgery | 0 | 0 | Not estimable | Not estimable | Not estimable |
| Transplant surgery | 1 | 22 | SMD 0.10 [-0.74, 0.94] | 0.82 | Not estimable |
| Gynaecological surgery | 0 | 0 | Not estimable | Not estimable | Not estimable |
| Trauma | 1 | 41 | SMD 0.06 [-0.56, 0.67] | 0.85 | Not estimable |
| Heterogeneity between subgroups (I^2^) = 6.1%, p=0.38 | | | | | |
| **Subgroup analysis: age** | | | | | |
| **Subgroup** | **Studies** | **Participants** | **Treatment effect [95% C.I.]** | **p value** | **Heterogeneity (I^2^)**  **(p value)** |
| **Self-reported quality of life** | | | | | |
| Under 65 | 8 | 581 | SMD 0.33 [0.13, 0.54] | 0.002 | 29%  (p=0.20) |
| Over 65 | 5 | 274 | SMD 0.17 [-0.15, 0.49] | 0.31 | 31%  (p=0.21) |
| Heterogeneity between subgroups (I^2^) = 0%, p=0.39 | | | | | |
| **Discharge to higher levels of care** | | | | | |
| Under 65 | 0 | 0 | Not estimable | Not estimable | Not estimable |
| Over 65 | 4 | 220 | OR 0.39 [0.15, 1.01] | 0.05 | 0%  (p=0.92) |
| Heterogeneity between subgroups (I^2^) = not estimable | | | | | |
| **Mortality at 30 days** | | | | | |
| Under 65 | 1 | 41 | OR 0.21 [0.02, 2.00] | 0.18 | Not estimable |
| Over 65 | 2 | 81 | OR 0.55 [0.06, 4.68] | 0.58 | 0%  (p=0.54) |
| Heterogeneity between subgroups (I^2^) = 0%, p=0.55 | | | | | |
| **Readmission rates** | | | | | |
| Under 65 | 1 | 32 | OR 0.47 [0.04, 5.73] | 0.55 | Not estimable |
| Over 65 | 1 | 61 | OR 0.34 [0.06, 1.94] | 0.23 | Not estimable |
| Heterogeneity between subgroups (I^2^) = 0%, p=0.85 | | | | | |
| **Length of hospital stay** | | | | | |
| Under 65 | 5 | 259 | SMD -0.19 [-0.44, 0.05] | 0.13 | 0%  (p=0.66) |
| Over 65 | 12 | 895 | SMD -0.43 [-0.91, 0.06] | 0.08 | 90%  (p<0.00001) |
| Heterogeneity between subgroups (I^2^) = 0%, p=0.39 | | | | | |
| **Subgroup analysis: timing of interventions** | | | | | |
| **Subgroup** | **Studies** | **Participants** | **Treatment effect [95% C.I.]** | **p value** | **Heterogeneity (I^2^)**  **(p value)** |
| **Self-reported quality of life** | | | | | |
| Pre-operative | 1 | 80 | SMD 0.48 [0.04, 0.93] | 0.03 | Not estimable |
| Peri-operative | 0 | 0 | Not estimable | Not estimable | Not estimable |
| Early post-operative | 7 | 441 | SMD 0.27 [-0.04, 0.58] | 0.09 | 56%  (p=0.03) |
| Late post-operative | 5 | 334 | SMD 0.19 [-0.03, 0.42] | 0.09 | 0%  (p=0.73) |
| Heterogeneity between subgroups (I^2^) = 0%, p=0.52 | | | | | |
| **Discharge to higher levels of care** | | | | | |
| Pre-operative | 0 | 0 | Not estimable | Not estimable | Not estimable |
| Peri-operative | 0 | 0 | Not estimable | Not estimable | Not estimable |
| Early post-operative | 1 | 15 | OR 0.33 [0.01, 9.57] | 0.52 | Not estimable |
| Late post-operative | 1 | 124 | OR 0.37 [0.12, 1.11] | 0.08 | Not estimable |
| Heterogeneity between subgroups (I^2^) = 0%, p=0.96 | | | | | |
| **Mortality at 30 days** | | | | | |
| Pre-operative | 0 | 0 | Not estimable | Not estimable | Not estimable |
| Peri-operative | 1 | 61 | OR 0.97 [0.06, 16.19] | 0.98 | Not estimable |
| Early post-operative | 2 | 61 | OR 0.22 [0.03, 1.43] | 0.11 | 0%  (p=0.94) |
| Late post-operative | 0 | 0 | Not estimable | Not estimable | Not estimable |
| Heterogeneity between subgroups (I^2^) = 0%, p=0.39 | | | | | |
| **Readmission rates** | | | | | |
| Pre-operative | 0 | 0 | Not estimable | Not estimable | Not estimable |
| Peri-operative | 1 | 61 | OR 0.34 [0.06, 1.94] | 0.23 | Not estimable |
| Early post-operative | 0 | 0 | Not estimable | Not estimable | Not estimable |
| Late post-operative | 1 | 32 | OR 0.47 [0.04, 5.73] | 0.55 | Not estimable |
| Heterogeneity between subgroups (I^2^) = 0%, p=0.85 | | | | | |
| **Length of hospital stay** | | | | | |
| Pre-operative | 1 | 53 | SMD-0.31 [-0.85, 0.23] | 0.26 | Not estimable |
| Peri-operative | 2 | 121 | SMD -0.01 [-0.37, 0.35] | 0.95 | 0%  (p=0.85) |
| Early post-operative | 10 | 807 | SMD-0.41 [-0.96, 0.14] | 0.14 | 92%  (p<0.00001) |
| Late post-operative | 1 | 21 | SMD -0.97 [-1.90, -0.05] | 0.04 | Not estimable |
| Heterogeneity between subgroups (I^2^) = 32%, p=0.22 | | | | | |
| **Subgroup analysis: cancer status** | | | | | |
| **Subgroup** | **Studies** | **Participants** | **Effect estimate** | **p value** | **Heterogeneity (I^2^)**  **(p value)** |
| **Self-reported quality of life** | | | | | |
| Cancer | 5 | 336 | SMD 0.14 [-0.08, 0.37] | 0.21 | 0%  (p=0.59) |
| No cancer | 8 | 519 | SMD 0.34 [0.10, 0.59] | 0.006 | 42%  (p=0.10) |
| Heterogeneity between subgroups (I^2^) = 29.1%, p=0.23 | | | | | |
| **Discharge to higher levels of care** | | | | | |
| Cancer | 1 | 15 | OR 0.33 [0.01, 9.57] | 0.52 | Not estimable |
| No cancer | 1 | 124 | OR 0.37 [0.12, 1.11] | 0.08 | Not estimable |
| Heterogeneity between subgroups (I^2^) = 0%, p=0.96 | | | | | |
| **Mortality at 30 days** | | | | | |
| Cancer | 1 | 61 | OR 0.97 [0.06, 16.19] | 0.98 | Not estimable |
| No cancer | 2 | 61 | OR 0.22 [0.03, 1.43] | 0.11 | 0%  (p=0.94) |
| Heterogeneity between subgroups (I^2^) = 0%, p=0.39 | | | | | |
| **Readmission rates** | | | | | |
| Cancer | 1 | 61 | OR 0.34 [0.06, 1.94] | 0.23 | Not estimable |
| No cancer | 1 | 32 | OR 0.47 [0.04, 5.73] | 0.55 | Not estimable |
| Heterogeneity between subgroups (I^2^) = 0%, p=0.85 | | | | | |
| **Length of hospital stay** | | | | | |
| Cancer | 6 | 291 | SMD -0.16 [-0.40, 0.09] | 0.12 | 6%  (p=0.38) |
| No cancer | 11 | 863 | SMD -0.39 [-0.89, 0.11] | 0.2 | 91%  (p<0.00001) |
| Heterogeneity between subgroups (I^2^) = 0%, p=0.85 | | | | | |
