# Supplementary material for: Interventions to prevent and treat sarcopenia in a surgical population: a systematic review and meta-analysis
Source: BJS Open. 2021 Jun 24;5(3):zraa069. doi: 10.1093/bjsopen/zraa069 (PMC8226286; doi:10.1093/bjsopen/zraa069)
Supplement: zraa069_Supplementary_Data [file zraa069_supplementary_data.zip › Supplement File 1.docx]

**Sarcopenia Systematic Review – Search Strategy**

Searches run on EMBASE through Ovid (13/12/19)

1. exp sarcopenia/
2. ((muscle or muscular) adj5 (atroph* or loss or fatigue or tonus)).ti,ab.
3. exp Dietary Proteins/
4. (protein* adj3 (diet* or supplement* or intake* or consum* or ingest*)).ti,ab.
5. exp exercise therapy/
6. ((train* or exercise*) adj5 (prehabilitation or rehabilitation or weight$ or resistance$)).ti,ab.
7. ((mineral$ or calcium or iron or magnesium or phosphorus or potassium or selenium or sodium or zinc) adj3 (diet* or supplement* or intake* or consum* or ingest*)).ti,ab.
8. (testosterone or androgen or growth hormone or GH).ti,ab.
9. ((hydroxymethylbutyrate or HMB) adj3 (diet* or supplement* or intake* or consum* or ingest*)).ti,ab.
10. ((25OH-vitaminD* or vitamin D or ascorbic acid or vitamin C) adj3 (diet* or supplement* or intake* or consum* or ingest*)).ti,ab.
11. or/1-2
12. or/3-10
13. 11 and 12
14. randomized controlled trial/
15. controlled clinical study/
16. placebo.ti,ab.
17. (compare or compared or comparison).ti.
18. ((evaluated or evaluate or evaluating or assessed or assess) and (compare or compared or comparing or comparison)).ab.
19. ((double or single or doubly or singly) adj (blind or blinded or blindly)).ti,ab.
20. parallel group$1.ti,ab.
21. ((assign$ or match or matched or allocation) adj5 (alternate or group$1 or intervention$1 or patient$1 or subject$1 or participant$1)).ti,ab.
22. (controlled adj7 (study or design or trial)).ti,ab.
23. trial.ti.
24. or/14-23
25. (rat or rats or mouse or mice or swine or porcine or murine or sheep or lambs or pigs or piglets or rabbit or rabbits or cat or cats or dog or dogs or cattle or bovine or monkey or monkeys or trout or marmoset$1).ti. and animal experiment/
26. (Animal/ or Animal experiment/) not (human experiment/ or human/)
27. 25 or 26
28. 24 not 27
29. 13 and 28

**1450**

Searches run on Medline through Ovid (13/12/19)

1. exp sarcopenia/
2. ((muscle or muscular) adj5 (atroph* or loss or fatigue or tonus)).ti,ab.
3. exp Dietary Proteins/
4. (protein* adj3 (diet* or supplement* or intake* or consum* or ingest*)).ti,ab.
5. exp exercise therapy/
6. ((train* or exercise*) adj5 (prehabilitation or rehabilitation or weight$ or resistance$)).ti,ab.
7. ((mineral$ or calcium or iron or magnesium or phosphorus or potassium or selenium or sodium or zinc) adj3 (diet* or supplement* or intake* or consum* or ingest*)).ti,ab.
8. (testosterone or androgen or growth hormone or GH).ti,ab.
9. ((hydroxymethylbutyrate or HMB) adj3 (diet* or supplement* or intake* or consum* or ingest*)).ti,ab.
10. ((25OH-vitaminD* or vitamin D or ascorbic acid or vitamin C) adj3 (diet* or supplement* or intake* or consum* or ingest*)).ti,ab.
11. or/1-2
12. or/3-10
13. 11 and 12
14. randomized controlled trial/
15. controlled clinical study/
16. placebo.ti,ab.
17. (compare or compared or comparison).ti.
18. ((evaluated or evaluate or evaluating or assessed or assess) and (compare or compared or comparing or comparison)).ab.
19. ((double or single or doubly or singly) adj (blind or blinded or blindly)).ti,ab.
20. parallel group$1.ti,ab.
21. ((assign$ or match or matched or allocation) adj5 (alternate or group$1 or intervention$1 or patient$1 or subject$1 or participant$1)).ti,ab.
22. (controlled adj7 (study or design or trial)).ti,ab.
23. trial.ti.
24. or/14-23
25. (rat or rats or mouse or mice or swine or porcine or murine or sheep or lambs or pigs or piglets or rabbit or rabbits or cat or cats or dog or dogs or cattle or bovine or monkey or monkeys or trout or marmoset$1).ti. and animal experiment/
26. (Animal/ or Animal experiment/) not (human experiment/ or human/)
27. 25 or 26
28. 24 not 27
29. 13 and 28

**843**

Searches run on CENTRAL (13/12/19)

1. MeSH descriptor: [Sarcopenia] explode all trees 354
2. ((muscle or muscular) NEXT5 (atroph* or loss or fatigue or tonus)) 92
3. MeSH descriptor: [Dietary Proteins] explode all trees 3884
4. (protein* NEXT3 (diet* or supplement* or intake* or consum* or ingest*)) 114
5. MeSH descriptor: [Exercise Therapy] explode all trees 12598
6. ((train* or exercise*) NEXT5 (prehabilitation or rehabilitation or weight$ or resistance$)) 173
7. ((testosterone or androgen or growth hormone or GH)):ti,ab,kw 19101
8. ((hydroxy methylbutyrate or HMB) NEXT3 (diet* or supplement* or intake* or consum* or ingest*)) 107
9. ((vitamin D or vitamin C) NEXT3 (diet* or supplement* or intake* or consum* or ingest*)) 109
10. #1 OR #2 446
11. {OR #3-#9} 35307
12. #10 AND #11 169

**54 Cochrane Reviews, 1 Cochrane Protocol, 114 Trials**

- **Search Results: 2537**
- **After Duplicates Removal: 1895**
